# Kinetic Mathematical Modeling of Oxidative Phosphorylation in Cardiomyocyte Mitochondria

**DOI:** 10.3390/cells11244020

**Published:** 2022-12-12

**Authors:** Wen-Wei Tseng, An-Chi Wei

**Affiliations:** 1 The Graduate Institute of Biomedical Electronics and Bioinformatics, National Taiwan University, Taipei 106, Taiwan; 2 Department of Electrical Engineering, National Taiwan University, Taipei 106, Taiwan

**Keywords:** oxidative phosphorylation, mitochondria, kinetic modeling, ATP, electron transport chain

## Abstract

Oxidative phosphorylation (OXPHOS) is an oxygen-dependent process that consumes catabolized nutrients to produce adenosine triphosphate (ATP) to drive energy-dependent biological processes such as excitation-contraction coupling in cardiomyocytes. In addition to in vivo and in vitro experiments, in silico models are valuable for investigating the underlying mechanisms of OXPHOS and predicting its consequences in both physiological and pathological conditions. Here, we compare several prominent kinetic models of OXPHOS in cardiomyocytes. We examine how their mathematical expressions were derived, how their parameters were obtained, the conditions of their experimental counterparts, and the predictions they generated. We aim to explore the general landscape of energy production mechanisms in cardiomyocytes for future in silico models.

## 1. Introduction

**Figure 1:**
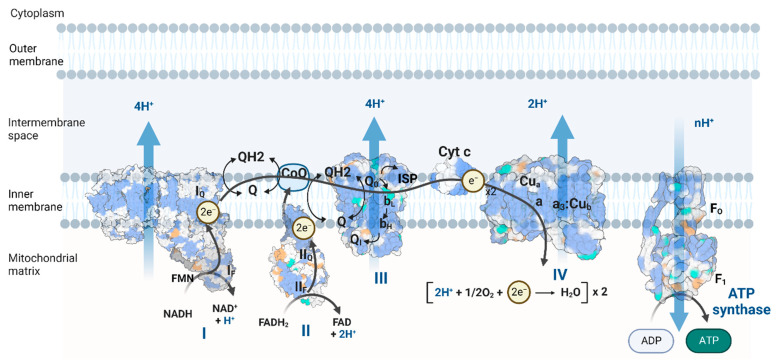
Schematic of OXPHOS, including the electron transport chain (ETC) and ATP synthase. The figure was adapted and created from “Electron Transport Chain”, by BioRender.com (accessed on 20 August 2022). Retrieved from https://app.biorender.com/biorender-templates (accessed on 20 August 2022).

The metabolic process of oxidative phosphorylation (OXPHOS) consumes chemical energy from catabolism to generate adenosine triphosphate (ATP), the energy currency of the cell. OXPHOS complexes sit on the inner mitochondrial membrane (IMM), consisting of electron transport chain (ETC) complexes I, II, III, and IV, as well as ATP synthase (complex V) [1] (Figure 1). The ETC complexes transfer electrons from reducing equivalents (NADH, FADH2) to oxygen molecules in controlled steps and extract the released chemical energy to pump protons out of the mitochondrial matrix, generating a proton electrochemical gradient called the proton motive force (pmf) that drives ATP synthase to produce ATP [2,3,4]. Cells can couple ATP hydrolysis to energy-consuming biological processes, e.g., muscle contraction, ion homeostasis, and macromolecule synthesis. OXPHOS energy generation is more efficient than that of anaerobic glycolysis. Mitochondrial OXPHOS produces approximately 30 ATP molecules for one glucose molecule, compared to two ATP molecules in glycolysis [5]. Thus, mitochondria are famous as “the powerhouse of the cell [6]”.

Oxygen is an excellent electron acceptor, with a redox potential of ~800 mV per electron under physiological conditions [7]. Nonetheless, some oxygen molecules are not reduced completely to water in the ETC. Instead, they become reactive oxygen species (ROS): superoxide, hydrogen peroxide, and hydroxyl radicals [8]. These highly reactive molecules can serve as a redox signaling mechanism from the mitochondria under physiological conditions [9,10,11]. However, excessive ROS can damage biomolecules, such as mitochondrial DNA (mtDNA), mitochondrial proteins for metabolism (e.g., aconitase and complex I), and the peroxidation of membrane lipids crucial for OXPHOS function [12,13]. The ROS scavenging systems include superoxide dismutase (SOD), cytochrome c, catalase, glutathione (GSH), and thioredoxin (Trx) systems [14,15].

Mitochondrial dysfunction is involved in a variety of pathological conditions, such as type 2 diabetes [16,17,18,19,20,21], liver toxicity [22,23], neurodegenerative diseases [24,25,26,27,28], arrhythmia, and heart failure [29,30,31,32,33,34]. In this review, we focus on the kinetic modeling of ATP generation in cardiomyocyte mitochondria because (i) many computational models and experimental data on cardiomyocyte mitochondria are available; (ii) cardiomyocytes rely on mitochondria-derived ATP for action potential firing, muscle contraction, and ion homeostasis in each heartbeat [35]. Mitochondria can take up to 30% of the cardiomyocyte volume [36]. However, cardiomyocytes maintain metabolite stability under varying workloads [37]; (iii) the OXPHOS system is interconnected with other metabolic pathways, such as the citric acid cycle, mitochondrial calcium handling, metabolite transfer, and ROS generation and scavenging. The interactions between these systems are related to cardiomyocyte pathologies such as altered calcium dynamics and electrophysiology, heart failure, ischemic-reperfusion injury, diabetic cardiomyopathy, and chemotherapy-induced cardiomyopathy [38]. Investigating the complex interactions and inner workings of OXPHOS and related metabolic systems using systems biology approaches and computer simulation models can complement the knowledge gathered from in vitro and in vivo studies, identify candidate mechanisms, and corroborate/propose novel hypotheses [39,40,41,42]. For these reasons, we review the mathematical modeling of OXPHOS complexes, focusing on cardiomyocyte bioenergetics models.

## 2. Description of Enzyme Reaction Rates

### 2.1. Law of Mass Action

The simplest way to describe a biochemical reaction rate is to assume a sufficiently large number of particles spreading evenly in a solution. Since the reaction rate is proportional to the number of particles colliding with each other, it is also proportional to the product of substrate concentrations to the power of their stoichiometric coefficients. For an elemental reversible reaction aA + bB = cP + dQ, the reaction rate in the PQ-forming direction can be described as:

$$v=k_{f}\left({\left[A\right]}^{a}{\left[B\right]}^{b}-{\left[P\right]}^{c}{\left[Q\right]}^{d}/K_{\mathrm{eq}}\right)$$

where the forward rate constant ($k_{f}$) is often an adjustable parameter determined by the literature and model fitting; the backward rate constant is constrained by the equilibrium constant ($K_{\mathrm{eq}}$). The reactants (A, B, P, and Q) can be substrates or different stages of the enzyme catalyzing the reaction. 

The law of mass action is the basis of the famous Michaelis-Menten enzyme kinetics and more complex schemes.

### 2.2. Quasi-Steady State Enzyme Kinetics

Michaelis-Menten kinetic expressions are derived from the law of mass action in enzyme catalysis reactions. Take an irreversible reaction S → P catalyzed by enzyme E as an example, assuming that the first substrate binding reaction is reversible and the second product formation reaction is irreversible.

E+S⇌ES→E+P

Suppose we treat the intermediate enzyme-substrate complex (ES) as the fast species and use the conservation relationship (Et = ES + E = constant) to constrain the free enzyme (E). In that case, the quasi-steady-state assumption reduces the two reactions into one by time-scale separation. The product formation rate is then described as a Michaelis-Menten hyperbolic function of the concentration of substrate S [43,44,45,46,47], where $V_{max}$ is the maximal reaction rate when the enzyme is saturated, and $K_{m}$ is the Michaelis constant, the concentration of S when the reaction rate reaches half of $V_{max}$.

$$V=V_{max}\frac{\left[S\right]}{\left[S\right]+K_{m}}$$

For enzyme reactions involving more than one substrate/product, more combinations, such as a random binding order, a compulsory binding order, ping-pong mechanisms, and dead-end intermediates, are possible [47,48]. The number of parameters and the complexity of the mathematical description increase as more binding and conversion scenarios are considered [43,49]. For simplicity, the enzyme and enzyme-substrate complexes are often treated as fast species, and their transitions are assumed to be in a quasi-steady-state (the rates of change of the transition states are set to zero). The state occupancies and the enzyme turnover rate can be solved systematically by linear algebra or by the Hill–King–Altman diagram method [50,51]. 

**Figure 2:**
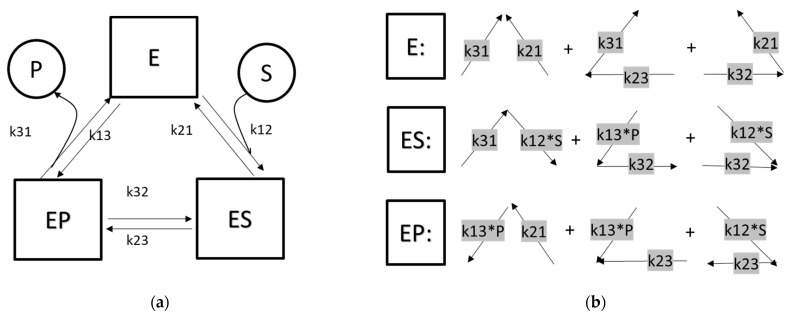
(**a**) Schematic of an enzyme-catalyzed reaction with three stages: the free enzyme (E, stage 1), the substrate-bound form (ES, stage 2), and the product-bound form (EP, stage 3). kij are rate constants transitioning from stage i to stage j. (**b**) The weight of each stage is the sum of the products of the transitional rates in the three connected graphs: Λ, <, and >.

For example, for a reversible enzyme-catalyzed reaction, S+E⇌ES⇌EP⇌E+P the reaction scheme can be drawn as in Figure 2. kij are rate constants for the enzyme state i transitioning to state j. After applying quasi-steady state approximation for the enzyme stages, the state occupancies of each stage can be solved by linear algebra.

$$\left[\begin{array}{ccc} -k_{12}S-k_{13}P & k_{21}\; & k_{31}\\ k_{12}S & -k_{21}-k_{23} & k_{32}\\ k_{13}P & k_{23} & -k_{31}-k_{32}\\ 1 & 1 & 1 \end{array}\right]\left[\begin{array}{c} E\\ ES\\ EP \end{array}\right]=\left[\begin{array}{c} \frac{dE}{dt}\\ \frac{dES}{dt}\\ \frac{dEP}{dt}\\ \sum E \end{array}\right]=\left[\begin{array}{c} 0\\ 0\\ 0\\ 1 \end{array}\right]$$

Alternatively, the state occupancies can be solved by the King–Altman–Hill diagram method [50]. This graph-based method first reduces the edges of the diagram to be a set of simple connected graphs without cycles. For example, the reaction cycle of Figure 2a can be broken down into three graphs: Λ, <, and > [50]. The weight of each stage ($w_{i}$) is the sum of the products of the transition rates indicated in Figure 2b.

$$\begin{array}{cc} v & =k_{23}f_{2}-k_{32}f_{3}\\ w_{1} & =k_{23}k_{31}+k_{32}k_{21}+k_{31}k_{21}\\ w_{2} & =k_{13}^{\prime }k_{32}+k_{23}k_{21}+k_{31}k_{12}^{\prime }\\ w_{3} & =k_{13}^{\prime }k_{23}+k_{12}^{\prime }k_{23}+k_{21}*k_{13}^{\prime }k_{23}\\ D & =w_{1}+w_{2}+w_{3}\\ f_{1} & =w_{1}/D\\ f_{2} & =w_{2}/D\\ f_{3} & =w_{3}/D\\ k_{12}^{\prime } & =k_{12}\left[\;S\right]\\ k_{13}^{\prime } & =k_{13}\left[P\right] \end{array}$$

The quasi-steady state reaction flux is then the product of the turnover rate (*v*) and the enzyme activity. For enzymes with more states and state transitions, a computer program called KAPattern [51] can automatically derive the weights of each state and the overall reaction rate.

## 3. Lumped Models of the Electron Transport Chain

A simplistic approach to describe reaction rates in the ETC is to treat the ETC complexes (I, II, III, and IV) as a single-unit proton pump.

Korzeniewski’s OXPHOS models [52,53] assumed that complex I and III reactions were close to equilibrium. The overall reaction rate of NADH reducing cytochrome c linearly depended on the thermodynamic span ($\Delta G_{13}$), the deviation between the current condition and the equilibrium. $\Delta G_{13}$ was determined by the ratios of NAD/NADH and oxidized/reduced cytochrome c couples, the mitochondrial membrane potential, and the proton motive force.

$$\begin{array}{cc} v_{C13} & =k_{\mathrm{redox}\;}\Delta G_{13}\\ \Delta G_{13} & =\Delta G_{C_{1}}+\Delta G_{C_{3}}\\ \Delta G_{C_{1}} & =E_{m,Q}-E_{m,N}-\frac{3}{2}\Delta p\\ \Delta G_{C_{3}} & =E_{m,c}-E_{m,Q}-\frac{2\Delta p+2\Delta \Psi }{2}\\ E_{m,N} & =E_{m,N0}+\frac{V_{T}}{2}\ln\left(\frac{\left[{\mathrm{NAD}}^{+}\right]}{\left[\mathrm{NADH}\right]}\right)\\ E_{m,c} & =E_{m,c0}+V_{T}\ln\left(\frac{\left[c^{3+}\right]}{\left[c^{2+}\right]}\right)\\ V_{T} & =\frac{RT}{F} \end{array}$$

**Figure 3:**
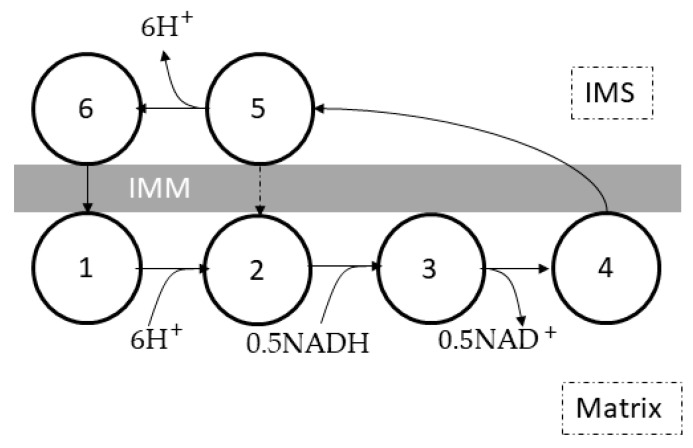
Schematic of the lumped complex I-III-IV model by Magnus and Keizer [54]. The six-state proton pump consists of proton translocation (5 → 6 → 1 → 2), NADH oxidation (2 → 3 → 4), and a slip reaction (5 → 2). Each cycle represents one electron transfer from NADH to oxygen, coupled with the translocation of 6 protons from the matrix to the intermembrane space (IMS). The arrows represent the forward reaction direction, although all reactions are reversible. Reprinted/adapted with permission from Ref. [54].

Magnus and Keizer [54] built a lumped complex I-III-IV ETC model (Figure 3) based on the six-state proton pump model by Pietrobon and Caplan [55] to study calcium dynamics and bioenergetics in mouse pancreatic beta-cell mitochondria. This model showed that the rates of proton translocation and oxygen consumption depended on the ratio of NADH to NAD and the mitochondrial membrane potential ($\Delta \Psi_{m}$).

$$\begin{array}{cc} J_{HR} & =6\rho_{res\;}\frac{f_{H}r_{a}f_{r}-\left(r_{a}+r_{b}\right)f_{\Psi }}{\left(1+r_{1}f_{r}\right)f_{B}+\left(r_{2}+r_{3}f_{r}\right)f_{\Psi }}\\ J_{O} & =0.5\rho_{res\;}\frac{\left(f_{H}r_{a}+r_{C1}f_{B}\right)f_{A}+f_{\Psi }\left(-r_{a}+r_{c2}f_{A}\right)}{\left(1+r_{1}f_{A}\right)f_{B}+\left(r_{2}+r_{3}f_{A}\right)f_{\Psi }}\\ f_{r} & =\sqrt{{\left[\mathrm{NADH}\right]}_{m}/{\left[{\mathrm{NAD}}^{+}\right]}_{m}}\\ f_{B} & =\exp\left(6\Delta \Psi_{B}/V_{T}\right)\\ f_{\Psi } & =\exp\left(6g\Delta \Psi_{m}/V_{T}\right)\\ f_{H} & =10^{6\Delta \mathrm{pH}}\\ V_{T} & =RT/F \end{array}$$

where $J_{HR}$ is the proton translocation rate, $J_{O}$ is the oxygen consumption rate, $V_{T}$ is the thermal voltage (~26.7 millivolts at 37 °C), $\rho_{res}$ is the concentration of the respiratory complex, $\Delta \Psi_{B}$ is the bulk phase boundary potential, g is the correction factor for $\Delta \Psi_{m}$, and $r_{a}$, $r_{b}$, $r_{c1}$, $r_{c2}$, $r_{1}$, $r_{2}$, and $r_{3}$ are constant parameters for the ETC. This lumped model did not include the ROS generation mechanism or the redox states of ubiquinone and cytochrome c pools.

The mitochondrial energetics model by Cortassa et al. [56] adapted Magnus and Keizer’s model [54] and replaced the dependence on the mitochondrial membrane potential ($\Delta \Psi_{m}$) with the proton motive force (pmf, Δp). The model added succinate oxidation flux via complexes II, III, and IV, depending on the pmf and the FADH2 to FAD ratio.

$$\begin{array}{cc} V_{He} & =6\rho^{\mathrm{res}\;}\frac{r_{a}v_{A}-\left(r_{a}+r_{b}\right)v_{H}^{6}}{\left(1+r_{1}v_{A}\right)v_{B}^{6}+\left(r_{2}+r_{3}v_{A}\right)v_{H}^{6}}\\ V_{He(F)} & =4\rho^{\mathrm{res}\;(F)}\frac{r_{a}v_{F}-\left(r_{a}+r_{b}\right)v_{H}^{4}}{\left(1+r_{1}v_{F}\right)v_{B}^{4}+\left(r_{2}+r_{3}v_{F}\right)v_{H}^{4}}\\ V_{O_{2}} & =0.5\rho^{\mathrm{res}\;}\frac{\left(r_{a}+r_{c1}v_{B}^{6}+r_{c2}v_{H}^{6}\right)v_{A}-r_{a}v_{H}^{6}}{\left(1+r_{1}v_{A}\right)v_{B}^{6}+\left(r_{2}+r_{3}v_{A}\right)v_{H}^{6}}\\ v_{A} & =K_{\mathrm{res}\;}\sqrt{\left[NADH\right]/\left[NAD^{+}\right]}\\ v_{F} & =K_{\mathrm{res}\;(F)}\sqrt{\left[FADH_{2}\right]/\left[FAD\right]}\\ v_{B} & =\exp\left(\Delta \Psi_{B}/V_{T}\right)\\ v_{H} & =\exp\left(g\Delta p/V_{T}\right)\\ \Delta p & =\Delta \Psi_{m}-2.303\Delta \mathrm{pH}\\ V_{T} & =RT/F \end{array}$$

where $V_{He}$ is the proton translocation rate from complex I-III-IV, $V_{He\left(F\right)}$ is the proton translocation rate from complex II-III-IV, $V_{O_{2}}$ is the oxygen consumption rate, Δp is the pmf, $\rho^{res\left(F\right)}$ is the concentration of complex II-III-IV, $V_{T}$ is the thermal voltage, $\rho_{res}$ is the concentration of the respiratory complex, $\Delta \Psi_{B}$ is the bulk phase boundary potential, g is the correction factor for $\Delta \Psi_{m}$, and $r_{a}$, $r_{b}$, $r_{c1}$, $r_{c2}$, $r_{1}$, $r_{2}$, and $r_{3}$ are constant parameters for the ETC.

## 4. Complex I

Mitochondrial complex I, also called NADH: ubiquinone oxidoreductase or type I NADH dehydrogenase, is the largest complex (with 46 subunits in mammalian mitochondria) in the ETC [57,58,59]. Its primary function is to transfer two electrons from one reduced nicotinamide adenine dinucleotide (NADH) molecule to one ubiquinone (coenzyme Q, denoted as Q) and pump four protons from the mitochondrial matrix to the intermembrane space. The chemical reaction is summarized as follows:

$$\mathrm{NADH}+Q+5H_{in}^{+}={\mathrm{NAD}}^{+}+{\mathrm{QH}}_{2}+4H_{out}^{+}$$

### 4.1. The Molecular Structure and Electron Transport in Complex I

The core molecular structure of complex I, sitting in the IMM, consists of one hydrophilic peripheral arm, protruding toward the mitochondrial matrix, and one hydrophobic membrane arm [58]. The peripheral arm conducts electron transfer from NADH to ubiquinone and consists of the NADH dehydrogenase module, the chain of iron-sulfur (FeS) clusters, and the ubiquinone reductase module. The membrane arm contains four parallel proton pumps; each translocates one proton once the bound ubiquinone is fully reduced and triggers a conformational change in the membrane arm. The redox process in complex I is reversible. Under a high pmf and a highly reduced Q pool with a large QH2 to Q ratio, the direction of the reaction favors QH2 oxidation, NAD+ reduction, and proton translocation from the IMS to the matrix. This is known as reverse electron transport (RET) [10]. Conversely, forward electron transport (FET) means that electrons flow ‘normally’ from complex I to ubiquinone.

The forward electron flow in complex I is NADH → FMN → FeS cluster chain (N3 → N1b → N4 → N5 → N6a → N6b → N2) → Q [57,58,59]. The NAD+/NADH couple can transfer only two electrons simultaneously, while the FeS cluster chain can handle only one electron at a time. Thus, FMN acts as a buffer between the NAD+/NADH couple and the FeS cluster chain because FMN can switch between three redox states: fully oxidized FMN, FMNH radical, and fully reduced FMNH2 [60]. Likewise, the ubiquinone molecule bound to the ubiquinone reductase receives electrons from the FeS clusters to form the ubisemiquinone radical (Q), and finally to the ubiquinol molecule (QH2). The rate of electron transport from NADH to FeS cluster N2 is much faster than the overall turnover rate of complex I (a time scale of 100 μs vs. 5 ms), with the FeS clusters mostly in the reduced state under physiological conditions, indicating that ubiquinone binding, reduction, and release, are rate-limiting steps [58]. The molecular structure of complex I also implies that the conformational changes induced by ubiquinone reduction couple the translocation of four protons from the matrix to the IMS [57].

### 4.2. Reactive Oxygen Species (ROS) Generation Mechanisms

Complex I is considered one of the major ROS producers in the mitochondria [12]. Several possible ROS-producing mechanisms are supported by experimental evidence in complex I [58,61,62,63]. Most studies recognize that the flavin site (IF) in the NADH dehydrogenase module and the quinone site (IQ) in the quinone reductase module are two major sources of ROS generation in complex I [64]. These two sites have different characteristics. ROS generation in the IF site is proportional to fully reduced flavin (FADH2) not bound by NAD/NADH [63]. The FeS cluster N1a can reduce the lifetime of superoxide-forming flavosemiquinone radical (FMNH•) [65]. ROS generation at the IF site is enhanced by blocking electron transport at the IQ site, for instance, by rotenone. ROS generation in the IQ site, on the other hand, is dependent on the fraction of bound semiquinone radicals, which is small during FET, but significant during RET. The inhibition of quinone reductase by rotenone decreases ROS generation during RET. Thus, whether rotenone enhances or inhibits ROS generation in complex I depends on several factors, including different substrates (glutamate/malate vs. succinate), the pmf, and the redox state of the ubiquinone pool, all of which determine the direction of complex I reaction. Kinetic modeling can help us elucidate the complicated dependence of the reaction rate on respiring substrates and the pmf in complex I.

### 4.3. Kinetic Models of Complex I

In Korzeniewski’s rate skeletal muscle OXPHOS models [53,66], the complex I reaction rate ($J_{C1}$) depended hyperbolically on its thermodynamic span ($\Delta E_{C1}$), the deviation of current condition from thermodynamic equilibrium.

$$J_{C1}=k_{C1}\frac{\Delta E_{C1}}{\Delta E_{C1}+K_{m}^{G1}}$$

Later, in a rat skeletal muscle OXPHOS model [67] and a rat cardiomyocyte OXPHOS model [68] showed a linear relationship between the complex I reaction rate ($J_{C1}$) and its thermodynamic span ($\Delta E_{C1}$).

$$J_{C1}=k_{C1}\Delta E_{C1}$$

The thermodynamic span ($\Delta E_{C1}$) can be derived from substrate (NAD/NADH and ubiquinone/ubiquinol couples) concentrations and the pmf (Δp).

$$\begin{array}{cc} \Delta E_{C_{1}} & =E_{m,Q}-E_{m,N}-\frac{4}{2}\Delta p\\ E_{m,N} & =E_{m,N0}+\frac{V_{T}}{2}\ln\left(\frac{\left[{\mathrm{NAD}}^{+}\right]}{\left[\mathrm{NADH}\right]}\right)\\ E_{m,Q} & =E_{m,Q0}+\frac{V_{T}}{2}\ln\left(\frac{\left[Q\right]}{\left[{\mathrm{QH}}_{2}\right]}\right)\\ V_{T} & =\frac{RT}{F} \end{array}$$

Beard’s OXPHOS model [69] also assumed that complex I operated near-equilibrium under physiological conditions and used a simple law of mass action descriptor for complex I reaction flux ($J_{C_{1}}$).

$$\begin{array}{cc} J_{C_{1}} & =X_{C_{1}}\left(K_{eq}^{C_{1}}\left[\mathrm{NADH}\right]\left[Q\right]-\left[{\mathrm{NAD}}^{+}\right]\left[{\mathrm{QH}}_{2}\right]\right)\\ K_{eq}^{C_{1}} & =\frac{{\left[H^{+}\right]}_{\mathrm{in}}^{5}}{{\left[H^{+}\right]}_{\mathrm{out}}^{4}}\exp\left(-\frac{\Delta_{r}G_{C_{1}}^{0}+4F\Delta \Psi_{m}}{RT}\right) \end{array}$$

where the enzyme activity ($X_{C_{1}}$) is an adjustable parameter fitted by experimental data of porcine cardiomyocyte mitochondria [70], and the equilibrium constant ($K_{eq}^{C_{1}}$) depends on the proton concentration, mitochondrial membrane potential ($\Delta \Psi_{m}$), and Gibbs free energy change ($\Delta_{r}G_{C_{1}}^{0}$) of the reaction. The descriptors for ROS generation were not included.

Heiske et al. [71] built an OXPHOS model, which described the complex I reaction rate ($v_{C_{1}}$) based on Henry–Michaelis–Menten kinetics.

$$\begin{array}{cc} v_{C_{1}} & =\frac{k_{f}\phi_{NADH}\phi_{Q}-k_{b}\phi_{NAD}\phi_{QH_{2}}}{\left(1+\phi_{NADH}+\phi_{NAD}\right)\left(1+\phi_{Q}+\phi_{QH_{2}}\right)}\\ \phi_{NADH} & =\frac{{\left[\mathrm{NADH}\right]}_{m}}{K_{m}^{NADH}},\;\;\phi_{NAD}=\frac{{\left[\mathrm{NAD}\right]}_{m}}{K_{m}^{NAD}}\\ \phi_{Q} & =\frac{\left[Q\right]}{K_{m}^{Q}},\;\;\phi_{QH_{2}}=\frac{\left[{\mathrm{QH}}_{2}\right]}{K_{m}^{QH_{2}}} \end{array}$$

where $k_{f}$ is the forward reaction constant, $k_{b}$ is the backward reaction constant, and $K_{m}^{x}$ is the apparent Michaelis constant for species *x* (NADH, NAD, QH2, or Q). The reaction constants and apparent Michaelis constants are thermodynamically constrained by the change in the Gibbs free energy in the reaction.

Markevich and Hoek’s mitochondrial ROS model [63] also included a complex I model using the law of mass action for its 17 reactions. Reactions of NAD/NADH and Q/QH2 couples, the IF (flavin) [60] and IQ (Q-reduction) site, and the FeS clusters of complex I were included to identify the source of ROS formation. They concluded that the majority of ROS production in complex I came from the IF site during FET and the IQ site during RET.

**Figure 4:**
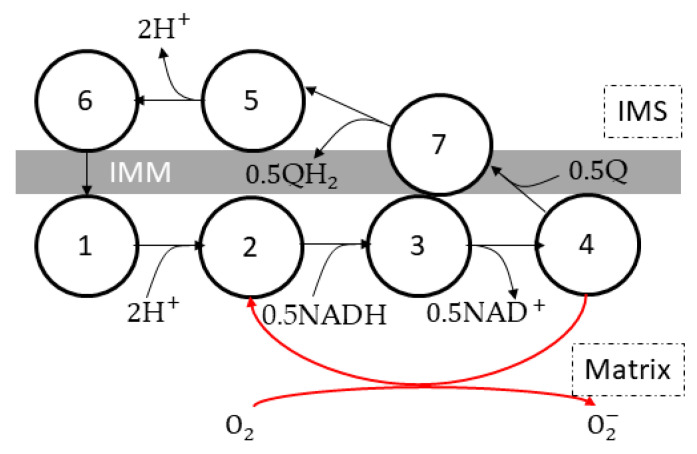
Schematic of the complex I model by Gauthier et al. [72]. The seven-state proton pump is adapted from the six-state proton pump model of Magnus and Keizer [54,55]. The model includes proton pumping (5 → 6 → 1→ 2), NADH oxidation (2 → 3 → 4), ubiquinone reduction (4 → 7 → 5), and superoxide generation (4 → 2). Each cycle represents one electron transfer from NADH to ubiquinone or oxygen. The arrows represent the forward reaction direction, although all reactions are reversible. Reprinted/adapted with permission from Ref. [62].

Gauthier et al. [62,72] built a seven-state complex I model (Figure 4) based on the Magnus–Keizer [54,55] six state proton pump model. The enzyme went through a cycle of NADH oxidation, ubiquinone reduction, and proton translocation (state 1→ 2 → 3 → 4 → 7 → 5 → 6 →1). A superoxide-generating pathway (state 4 → 2) was included.

**Figure 5:**
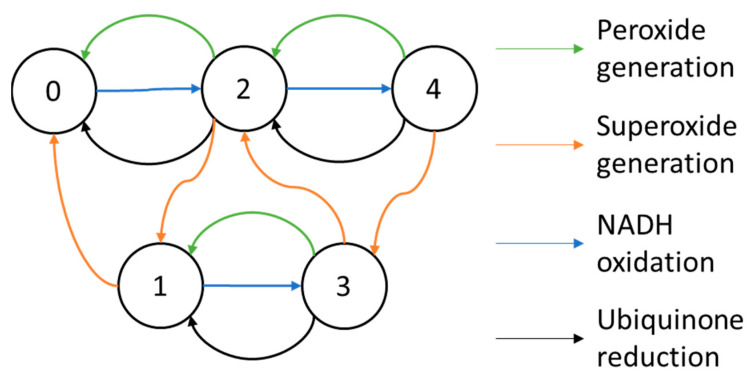
Schematic of the complex I model by Bazil et al. [61]. The 5 states represent the number of electrons held by complex I. The model includes peroxide generation reactions (green arrows) taking two electrons from complex I to oxygen, superoxide generation reactions (orange arrows) taking one electron from complex I to oxygen, NADH oxidation reactions (blue arrows) taking two electrons from NADH to complex I, and finally ubiquinone reduction reactions (black arrows) taking two electrons from complex I to ubiquinone, coupled with 4-proton translocation. The figure was reprinted/adapted with permission from Ref. [61].

The complex I model by Bazil et al. [61] includes five states by the number of electron(s) the complex holds (Figure 5). They assumed that the electron transfer inside complex I was much faster than reactions with substrates; electrons were distributed in steady-state across the redox centers: FMN, FeS cluster N2, and the Q-site. To eliminate internal state variables, the state transitions were also assumed to be in a quasi-steady state. The enzyme turnover and ROS generation rates were functions of the pmf and substrate concentrations. The kinetic parameters were fitted by the kinetic data and the ROS data from bovine heart mitochondria and constrained by thermodynamics in redox chemistry. They reproduced the reaction rates between NAD/NADH and Q/QH2 couples and the ROS production rates of superoxide and hydrogen peroxide.

## 5. Complex II

### 5.1. Molecular Structure and Reaction Mechanism

Mitochondrial complex II is also called succinate dehydrogenase (SDH), or succinate-coenzyme Q reductase (SQR). Complex II couples the citric acid cycle (CAC) with the ETC by transferring electrons from succinate to ubiquinone (Q), forming fumarate and ubiquinol (QH_2_) [73]:

Succinate + Q = fumarate + QH_2_

The molecular structure of complex II consists of four subunits: SDHA (succinate dehydrogenase site, with FAD as the prosthetic group), SDHB (FeS clusters), SDHC and SDHD (ubiquinone reductase site) [74]. The electron transfer inside complex II is summarized as follows:

Succinate ↔ FAD ↔ FeS clusters ↔ Q

In the forward direction, succinate is oxidized to fumarate, and Q is reduced to QH_2_. Nevertheless, the reaction is reversible, depending on the concentrations of succinate, fumarate, Q, and QH_2_. Significant succinate accumulation is found in ischemic cells and the strong forward reaction upon reoxygenation is responsible for RET-related ROS generation and ischemic-reperfusion injury [9,10,75]. Complex II activity and ROS generation from RET are inhibited by oxaloacetate and malonate [76].

### 5.2. Kinetic Models of Complex II

In the guinea pig cardiomyocyte mitochondrial model by Cortassa et al. [56], SDH irreversibly oxidized succinate (SUC) into fumarate, and was inhibited by oxaloacetate (OAA) and fumarate (FUM). However, in this model, succinate oxidation was not coupled to proton pumping in the ETC.

$$V_{SDH}=\frac{k_{cat}E_{T}\left[\mathrm{SUC}\right]}{\left[\mathrm{SUC}\right]+K_{m}\left(1+\frac{\left[\mathrm{OAA}\right]}{K_{i}^{OAA}}\right)\left(1+\frac{\left[\mathrm{FUM}\right]}{K_{i}^{FUM}}\right)}$$

where $k_{cat}$ is the catalytic constant, $E_{T}$ is the SDH concentration, $K_{m}$ is the Michaelis constant for succinate, $K_{i}^{OAA}$ is the inhibition constant for OAA, and $K_{i}^{FUM}$ is the inhibition constant for fumarate.

Wei’s acid-base equilibria model [77] (also used in [14]) coupled ETC and CAC reactions by assuming that the FADH2 to FAD ratio in the Cortassa et al. model [56] was determined by the succinate to fumarate ratio. The reaction rate was also inhibited by OAA. However, because complexes II, III, and IV were lumped, there were no reactions of the ubiquinone/ubiquinol couple.

$$\begin{array}{cc} V_{He(F)} & =4\rho^{\mathrm{res}(F)}\frac{r_{a}v_{F}-\left(r_{a}+r_{b}\right)v_{H}}{\left(1+r_{1}v_{F}\right)v_{B}+\left(r_{2}+r_{3}v_{F}\right)v_{H}}\frac{K_{i}}{\left[OAA\right]+K_{i}}\\ V_{O_{2}SDH} & =0.5\rho^{\mathrm{res}\;}\frac{\left(r_{a}+r_{C1}v_{B}+r_{C2}v_{H}\right)v_{A}-r_{a}v_{H}}{\left(1+r_{1}v_{A}\right)v_{B}+\left(r_{2}+r_{3}v_{A}\right)v_{H}}\frac{K_{i}}{\left[OAA\right]+K_{i}}\\ v_{F} & =\frac{K_{\mathrm{res}\;}^{\mathrm{SDH}\;}}{P_{\mathrm{SUC}\;}}\sqrt{\left[\;\mathrm{Succinate}\;]/[\;\mathrm{Fumarate}\;\right]}\\ v_{B} & =\exp\left(4\Delta \Psi_{B}/V_{T}\right)\\ v_{H} & =\exp\left(4g\Delta p/V_{T}\right)\\ V_{T} & =RT/F\\ \Delta p & =\Delta \Psi_{m}-2.303\Delta \mathrm{pH} \end{array}$$

where $V_{He\left(F\right)}$ is the proton translocation rate, $V_{O_{2}SDH}$ is the oxygen consumption rate, Δp is the pmf, $\rho^{res\left(F\right)}$ is the concentration of complex II-III-IV, $K_{res}^{SDH}$ is the equilibrium constant of succinate oxidation, $P_{SUC}$ is the binding polynomial for unprotonated succinate, $K_{i}$ is the inhibition constant for OAA, $\Delta \Psi_{B}$ is the bulk phase boundary potential, g is the correction factor for $\Delta \Psi_{m}$, and $r_{a}$, $r_{b}$, $r_{c1}$, $r_{c2}$, $r_{1}$, $r_{2}$, and $r_{3}$ are constant parameters for the ETC (complexes II, III, and IV).

Demin’s OXPHOS models [62,78,79] assumed succinate excess. The reaction rate of SDH depended hyperbolically on the fraction of oxidized ubiquinone in the Q pool.

$$\begin{array}{cc} J_{C2} & =\frac{V_{max}f_{Q}}{f_{Q}+K_{m}^{Q}}\\ f_{Q} & =\frac{\left[Q\right]}{\left[Q\right]+\left[{\mathrm{QH}}_{2}\right]} \end{array}$$

The complex II formulation in the hepatocyte mitochondria model by Mogilevskaya et al. [80] used random order rapid equilibrium Bi-Bi enzyme kinetics. The model was used to study salicylate toxicity to hepatocytes by inhibiting CAC activity.

$$\begin{array}{cc} J_{SDH} & =\left[\mathrm{SDH}\right]\frac{k_{f}AB-k_{b}PQ}{1+A+\alpha B+AB+P+\beta Q+PQ}\\ A & =\frac{\left[\mathrm{Suc}\right]}{K_{Esuc}},\;\;B=\frac{\left[Q\right]}{K_{m}^{Q}},\;\;\alpha =\frac{K_{m}^{SUC}}{K_{Esuc}}\\ P & =\frac{\left[\mathrm{Fum}\right]}{K_{Efum}},\;\;Q=\frac{\left[{\mathrm{QH}}_{2}\right]}{K_{m}^{QH_{2}}},\;\;\beta =\frac{K_{m}^{FUM}}{K_{Efum}} \end{array}$$

The mitochondrial OXPHOS model by Wu et al. [81,82,83] used the Theorell–Chance Bi-Bi mechanism for SDH. Inhibition by oxaloacetate was also included.

$$\begin{array}{cc} V_{SDH} & =\frac{V_{f}\cdot a\cdot b-V_{b}\cdot p\cdot q}{\alpha_{i}+a+\alpha_{i}b\frac{K_{mA}}{K_{iA}}+\alpha_{i}p\frac{K_{mQ}}{K_{\mathrm{iQ}\;}}+q+ab+bq\frac{K_{mA}}{K_{iA}}+ap\frac{K_{mQ}}{K_{iQ}}+pq}\\ a & =\frac{\left[\mathrm{SUC}\right]}{K_{iA}},\;b=\frac{\left[Q\right]}{K_{mB}},\;p=\frac{\left[{\mathrm{QH}}_{2}\right]}{K_{mP}},\;q=\frac{\left[\mathrm{FUM}\right]}{K_{iQ}}\\ \alpha_{i} & =\frac{1+\frac{\left[\mathrm{OAA}\right]}{K_{i}^{OAA}}+\frac{\left[\mathrm{SUC}\right]}{K_{a}^{SUC}}+\frac{\left[\mathrm{FUM}\right]}{K_{a}^{FUM}}}{1+\frac{\left[\mathrm{SUC}\right]}{K_{a}^{SUC}}+\frac{\left[\mathrm{FUM}\right]}{K_{a}^{FUM}}} \end{array}$$

**Figure 6:**
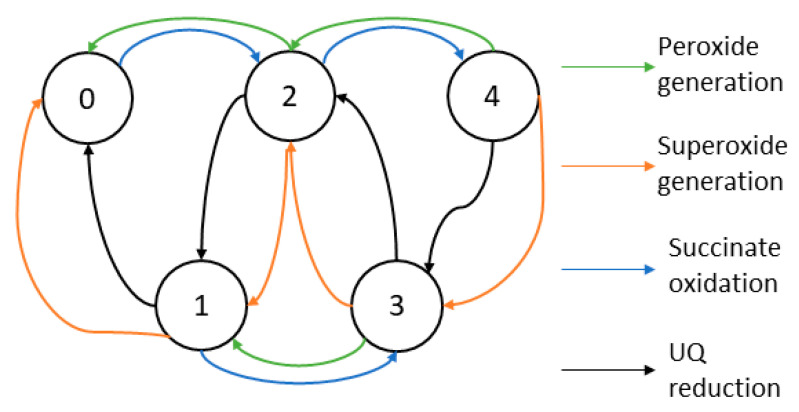
Schematic of the complex II model by Manhas et al. [84]. The 5 states represent the number of electrons in complex II. The model includes peroxide generation reactions (green arrows) taking two electrons from complex II to oxygen, superoxide generation reactions (orange arrows) taking one electron from complex II to oxygen, succinate oxidation reactions (blue arrows) taking two electrons from NADH to complex II, and finally ubiquinone reduction reactions (black arrows) taking one electron from complex II to ubiquinone. The figure was reprinted/adapted from Ref. [84].

Manhas et al. [84] devised a five-state complex II model to study how substrates and inhibitors affect its reaction rate and ROS generation (Figure 6). The authors assumed that electron transport within complex II is much faster than substrate binding (succinate, fumarate, Q/QH2 couple, and oxygen). The five states represent how many electrons are held by complex II, ranging from zero to five; the state transitions represent reactions between complex II and its substrates. The thermodynamically constrained parameters were fitted against various datasets from bovine, pig, and guinea pig heart mitochondria experiments. The authors reproduced the result of succinate oxidation kinetics with and without inhibitors (atpenin and malonate) and ROS production rates from complex II. The authors concluded that the primary source of ROS is the [3Fe-4S] iron-sulfur complex under most physiological conditions.

Markevich et al. [85] developed a complex II model using mass action kinetics, which included substrate binding and electron transport processes with a total of 31 reactions. The parameters were fitted against experimental data from bovine and rat heart mitochondria. The authors also investigated the difference between intact and disintegrated complex II, where SDHA/SDHB subunits were separated from SDHC/SDHD subunits. The authors concluded that in disintegrated complex II, Q-binding site inhibition, and complex III inhibition had a high-amplitude bell-shaped dependence on succinate in ROS generation. In contrast, intact complex II under physiological conditions had a hyperbolic dependence on succinate in ROS generation.

## 6. Complex III

Respiratory complex III is also called the cytochrome bc1 complex, coenzyme Q:cytochrome c—oxidoreductase. It transfers electrons from ubiquinol (QH_2_) to cytochrome c (cytc) by the following reaction:

$$2{\mathrm{QH}}_{2}\left(p\right)+Q\left(n\right)+2{\mathrm{cytc}}^{3+}+2H_{\mathrm{in}}^{+}=2Q\left(p\right)+{\mathrm{QH}}_{2}\left(n\right)+2{\mathrm{cytc}}^{2+}+4H_{out}^{+}$$

### 6.1. Molecular Structure and Reaction Mechanism

Complex III translocates protons outward via Mitchell’s Q-cycle [86]. The first and rate-limiting reaction is ubiquinol oxidation to ubiquinone at the Qo (outer) site. The two electrons from ubiquinol bifurcate toward two paths. One electron goes through the high potential pathway (Qo → iron-sulfur protein (ISP) → cyt c1 → cyt c) to reduce cytochrome c; the other goes through the low potential pathway (Qo → cyt bL → cyt bH → Qi) to reduce ubiquinone at the Qi (inner) site. Two ubiquinol molecules oxidized at the Qo site complete a full cycle and release four protons to the intermembrane space. Meanwhile, one ubiquinone molecule at the Qi site is reduced to ubiquinol, absorbing two protons from the mitochondrial matrix. Overall, the Q-cycle translocates two charges for every ubiquinol oxidized at the Qo site with two electrons transferred [87].

On the inner mitochondrial membrane, complex III monomers form dimers, allowing electron transfer between their two cyt bL centers. However, electron transfer between monomers has been considered nonsignificant; the dimer acts as two independent monomers under physiological conditions [88,89,90].

Commonly used complex III inhibitors in experiments include antimycin A (AA) (blocking the Qi site), stigmatellin (completely blocking the Qo site), and myxothiazol (partially blocking the Qo site) [87,91,92].

### 6.2. Reactive Oxygen Species Generation Mechanisms

In addition to complex I, complex III is another major source of mitochondrial-derived reactive oxygen species. (mtROS) Semiquinone radicals (SQ) at the Qo site can reduce oxygen to form superoxide. There are two mechanisms for SQ formation, the semiforward and the semireverse mechanisms. The semiforward mechanism states that after ubiquinol (QH2) at the Qo site donates one electron to the iron-sulfur protein (ISP), the remaining SQ intermediate reduces oxygen to form superoxide. [89] In contrast, the semireverse mechanism argues that the SQ intermediate is too unstable to stay and play a significant role in superoxide generation. Instead, QH2 donates all its electrons downstream, one to ISP and the other to bL. [89] Superoxide forms when bL is highly reduced due to high pmf or the downstream pathway is blocked by inhibitors such as antimycin A. An electron can jump from reduced bL to a ubiquinone molecule at the Qo site, forming a transient SQ intermediate, which reduces oxygen to superoxide [87,93]. Both mechanisms of superoxide generation are strongly dependent on the pmf, but the semireverse mechanism implies that the rate of superoxide generation is maximized when the ubiquinone pool is moderately reduced, which is more consistent with experimental findings on antimycin-induced ROS generation from complex III [87]. Complex III releases superoxide to both sides of the IMM [94].

### 6.3. Kinetic Models of Complex III

In Korzeniewski’s rate skeletal muscle OXPHOS models [53,66], the complex III reaction rate ($J_{C3}$) depended hyperbolically on its thermodynamic span ($\Delta E_{C3}$), the deviation of current condition from thermodynamic equilibrium.

$$J_{C3}=k_{C3}\frac{\Delta E_{C3}}{\Delta E_{C3}+K_{m}^{G3}}$$

A rat skeletal muscle OXPHOS model [67] and a rat cardiomyocyte OXPHOS model [68] by Korzeniewski et al. showed a linear relationship between the complex I reaction rate ($J_{C3}$) and its thermodynamic span ($\Delta E_{C3}$).

$$J_{C3}=k_{C3}\Delta E_{C3}$$

The thermodynamic span ($\Delta E_{C3}$) can be derived from substrate (ubiquinone/ubiquinol and oxidized/reduced cytochrome c couples) concentrations, the mitochondrial membrane potential, and the pH difference across the IMM (embedded in the pmf, Δp).

$$\begin{array}{cc} \Delta G_{C_{3}} & =E_{m,c}-E_{m,Q}-\frac{2\Delta p+2\Delta \Psi }{2}\\ E_{m,Q} & =E_{m,Q0}+\frac{V_{T}}{2}\ln\left(\frac{\left[Q\right]}{\left[{\mathrm{QH}}_{2}\right]}\right)\\ E_{m,c} & =E_{m,c0}+V_{T}\ln\left(\frac{\left[c^{3+}\right]}{\left[c^{2+}\right]}\right)\\ V_{T} & =\frac{RT}{F} \end{array}$$

Beard’s OXPHOS model [69] assumed that the complex III reaction operated near equilibrium and used the law of mass action for the reaction rate. Activation from inorganic phosphate (${\left[\mathrm{Pi}\right]}_{m}$) was added to explain the data in a mitochondrial experiment [70]. However, a later study [95] found that complex III activity was not enhanced directly by inorganic phosphate. The apparent activation by inorganic phosphate was likely due to associated substrate transport and nonsteady-state experimental conditions.

$$\begin{array}{cc} J_{C_{3}} & =X_{C_{3}}\frac{1+\frac{{\left[\mathrm{Pi}\right]}_{m}}{K_{p_{1}}}}{1+\frac{{\left[\mathrm{Pi}\right]}_{m}}{K_{p_{2}}}}\left(\left[c^{2+}\right]\sqrt{K_{eq}^{C_{3}}\left[{\mathrm{QH}}_{2}\right]}-\left[c^{3+}\right]\sqrt{\left[Q\right]}\right)\\ K_{eq}^{C_{3}} & =\frac{{\left[H^{+}\right]}_{\mathrm{in}}^{2}}{{\left[H^{+}\right]}_{\mathrm{out}}^{4}}\exp\left(-\frac{\Delta_{r}G_{C_{3}}^{0}+2F\Delta \Psi_{m}}{RT}\right) \end{array}$$

Heiske et al. [71] built an OXPHOS model, where they described the complex III reaction rate ($J_{c3}$) based on Michaelis–Menten kinetics. The apparent activation by inorganic phosphate was inherited from Beard’s OXPHOS model [69].

$$\begin{array}{cc} J_{C_{3}} & =\frac{k_{f}\phi_{QH_{2}}\phi_{c_{3}}^{2}-k_{b}\phi_{Q}\phi_{c_{2}}^{2}}{\left(1+\phi_{QH_{2}}+\phi_{Q}\right){\left(1+\phi_{c_{2}}+\phi_{c_{3}}\right)}^{2}}\frac{\left[\mathrm{Pi}\right]}{\left[\mathrm{Pi}\right]+K_{A}}\\ \phi_{Q} & =\frac{\left[Q\right]}{K_{m}^{Q}},\;\;\phi_{QH_{2}}=\frac{\left[{\mathrm{QH}}_{2}\right]}{K_{m}^{QH_{2}}}\\ \phi_{c_{3}} & =\frac{\left[c_{3}^{+}\right]}{K_{m}^{c_{3}}},\;\;\phi_{c_{2}}=\frac{\left[c_{2}^{+}\right]}{K_{m}^{c_{2}}} \end{array}$$

where $k_{f}$ is the forward reaction constant, $k_{b}$ is the backward reaction constant, and $K_{m}^{x}$ is the apparent Michaelis constant for species *x* (ubiquinone, ubiquinol, reduced and oxidized cytochrome c). The reaction constants and apparent Michaelis constants are thermodynamically constrained by the change in Gibbs free energy in the reaction.

Demin et al. [79] modeled Mitchell’s Q-cycle using the law of mass action. The system included ubiquinone diffusion on the IMM and electron transfer between ubiquinone, complex III redox centers (ISP, cyt c1, bL, and bH), and cyt c. The model used the semiforward mechanism for ROS generation; in the model, superoxide was generated from the oxygen oxidizing Qo site ubisemiquinone radical. The model consisted of two variants: the minimal and channeled models. The minimal model treated the Qo site ubisemiquinone radical as part of the Q pool, not binding to complex III. In the channeled model, on the other hand, Qo site ubisemiquinone radicals were bound to complex III, next to either ISP or heme bL. The superoxide generation rate showed exponential dependence on the mitochondrial membrane potential in the minimal model but sigmoid dependence in the channeled model. The minimal model was later integrated into the redox balance model by Gauthier et al. [62].

Demin et al. [78] also built a newer model by coupling the redox center states of ISP, cyt bL, and cyt bH to ensure electron bifurcation. The semiquinone radical at the Qo site remained bound until bL was oxidized by bH. The locking mechanism prevented both electrons from ubiquinol from going through ISP and bypassing the Q-cycle. 

Selivanov’s complex III model [96] included all possible combinations of binding and redox states, including ubiquinone (at both the p-side and n-side), cytochromes bL and bH, ISP, and cytochrome c1, creating a 400-state ODE model. They found that there are two steady-state branches in complex III in simulations and experiments. Increasing the succinate supply or reducing the oxygen concentration switched complex III from a low ROS-generating mode to a high ROS-generating mode, which could explain the increased ROS production from ischemia-reperfusion.

Guillaud et al. [97] constructed a 16-state complex III model in which superoxide was produced via the semireverse mechanism by reduced cytochrome bL and oxidized ubiquinone. Mass action kinetics described the transition between the redox states of ISP, cyt bL, cyt bH, and the Qi site. The model reproduced the bell-shaped dependence of ROS generation on the fraction of reduced quinone in the experiments.

Markevich and Hoek [63] developed a complex III model using mass action kinetics to test competing ROS generation mechanisms under a variety of proton motive forces, substrate levels, and inhibitors. They concluded that the late dissociation of ISP, that is, ISP leaving cyt bL after ubiquinone, is more consistent with experimental data.

**Figure 7:**
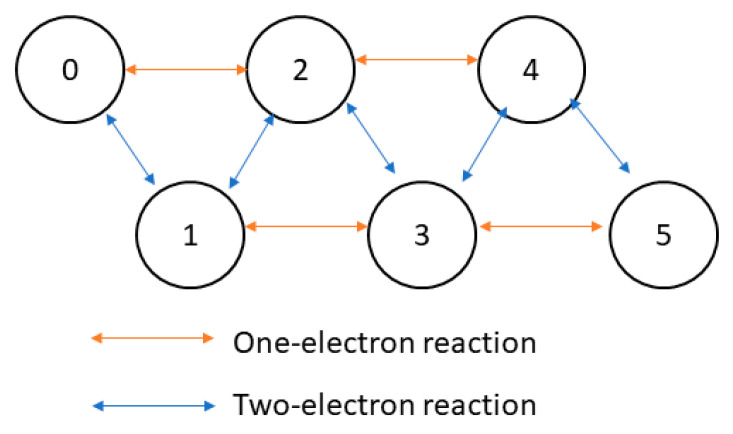
Schematic of the dimeric complex III model by Bazil et al. [99]. The 6 states represent the number of electrons in complex III. The model includes one-electron transfer reactions (orange arrows): ubiquinol oxidation at the Qo site (one electron goes to heme bL, the other goes through ISP, cytochrome c1, and finally to cytochrome c) as well as oxygen reduction to superoxide. The model also has a two-electron transfer reaction: Q reduction at the Qi site. The figure was reprinted/adapted from Ref. [99].

Bazil et al. [98] developed an algebraic descriptor of complex III to reproduce experimental results of electron transport, ROS generation, and bistability. The model used a six-state scheme to represent the number of electrons on the Qo and Qi sites. Kinetic rate constants were determined by experimental data, pH, the pmf, and changes in the Gibbs free energy for state transitions. The net turnover flux was derived from state occupancies at steady state, obtained by solving a linear system of equations. Later, a complex III functional dimer model [99] was built upon a similar principle (Figure 7). The dimer model had six-states representing zero to five electrons shared by the redox centers. The dimer model not only reproduced bistability in electron transfer and ROS generation but also showed that low-dose antimycin partially blocking the Qi site stimulates cytochrome c reduction, which can only be explained via a dimer model without using a different parameter set. The dimer model also revealed that ROS were generated predominantly via the semireverse mechanism by reduced cytochrome bL under most conditions. ROS generation from the semiforward mechanism (by semiquinone radical) became significant only with a highly reduced ubiquinone pool under antimycin blocking the Qi site.

## 7. Complex IV

Respiratory complex IV, also called cytochrome c oxidase (CcO), is the terminal enzyme of the ETC, receiving electrons from cytochrome c and catalyzing the reduction of oxygen to water [100]. For every oxygen molecule reduced, four cytochrome c molecules are oxidized, and eight positive charges are translocated outward against the mitochondrial membrane potential.

$$4{\mathrm{cytc}}^{2+}+O_{2}+8H_{\mathrm{in}}^{+}\to 4{\mathrm{cytc}}^{3+}+2H_{2}O+4H_{out}^{+}$$

### 7.1. Molecular Structure and Reaction Mechanism

In complex IV, electrons flow from cytochrome c → CuA → cytochrome a → cytochrome a3-CuB binuclear center (BNC) to oxygen. Because every cytochrome c molecule carries only one electron, it requires four reduced cytochrome c molecules to fully reduce one oxygen molecule to water. 

The active site of complex IV to oxygen is the BNC and the catalytic cycle consists of two phases: the eu-oxidase phase and the peroxidase phase [100]. In the eu-oxidase phase, an oxygen molecule binds doubly reduced BNC and is reduced to peroxide. In the peroxidase phase, BNC receives two electrons, fully reducing peroxide to water. Under physiological conditions, complex IV operates continuously and each electron transfer from cytochrome c is coupled to the outward translocation of one proton [100,101,102]. However, slip reactions (electron transfer without proton pumping) have been reported in isolated complex IV [103], with two protons instead of four being pumped per oxygen molecule reduced. It is also hypothesized that under a high pmf, complex IV trades proton pumping efficiency for a higher enzyme turnover via the slip pathway. 

The BNC also evens out the redox potential changes for each electron transfer, keeping the midpoint potential for oxygen reduction at approximately −800 mV per electron [102]. The tight interactions between BNC and oxygen ligands also minimize ROS generation during the reaction [12,104]. 

Complex IV activity is inhibited by nitric oxide (NO) [105,106], cyanide (CN^−^) [107], carbon monoxide (CO) [108], hydrogen sulfide (H_2_S) [109], azide (N_3_^−^) [110], and formate [111], the toxicity of which can block the electron transport chain and ATP synthesis. Among complex IV inhibitors, nitric oxide (NO) is the most physiologically relevant because under physiological conditions, a substantial fraction (up to half) of complex IV activity is inhibited by NO [105,106]. NO inhibits complex IV in two modes: competitive inhibition with oxygen binding and uncompetitive inhibition in which NO is reduced to nitrite. NO binding to complex IV is also sensitive to near infrared and red photons, causing the dissociation of NO, and freeing up available complex IV for OXPHOS [106,112].

### 7.2. Kinetic Models of Complex IV

The early OXPHOS model by Korzeniewski and Froncisz [52] included a three-state (A1, A2, and A3) complex IV reaction cycle [113] that depended on the phosphorylation potential.

$$\begin{array}{cc} v_{1} & =k_{1}\left[A_{2a}\right]\left[c^{2+}\right]\\ v_{-1} & =k_{-1}\left[A_{3}\right]\left[c^{3+}\right]\\ v_{2} & =k_{2}\left[A_{3}\right]\left[O_{2}\right]\\ v_{-2} & =k_{-2}\left[A_{1a}\right]\\ v_{4} & =\left(k_{4a}\left[A_{1a}\right]+k_{4b}\left[A_{1b}\right]\right)\left[c^{2+}\right]\\ \left[A_{2a}\right] & =\left[A_{2}\right]\frac{1}{1+g}\\ g & ={\left(\frac{\left[c^{3+}\right]}{\left[c^{2+}\right]}\right)}^{2}\left(\frac{AAP}{K_{5}}\right)\\ \left[A_{1a}\right] & =\left[A_{1}\right]\frac{1}{1+K_{3}}\\ \left[A_{1b}\right] & =\left[A_{1}\right]\frac{K_{3}}{1+K_{3}}\\ K_{3} & =Q\exp\left(\frac{\left.E_{m,a_{3}}-E_{m,cu}\right)F}{RT}\right)\\ E_{m,a_{3}} & =E_{m_{0},a_{3}}-\frac{RT}{F}\cdot \ln\left(\frac{8\times 10^{-7}+AAP}{5\times 10^{-3}+AAP}\right)\\ AAP & =\frac{\left[\mathrm{ATP}\right]}{\left[\mathrm{ADP}][\mathrm{Pi}\right]} \end{array}$$

The reaction rate depended on the ratio of oxidized and reduced cytochrome c couple, the oxygen concentration, and the phosphorylation potential term (AAP), which is the quotient of ATP by ADP and inorganic phosphate concentrations. 

In Korzeniewski’s later OXPHOS models for hepatocytes [114] and myocytes [53], the descriptors for complex IV were simplified and dependent on the pmf. The reaction rate depended on the oxygen concentration, the reduced cytochrome c concentration ($c^{2+}$), and the availability of reduced cytochrome a3 ($a^{2+}$). $a^{2+}$ was assumed in rapid equilibrium and was derived from the ratio of oxidized and reduced cytochrome c couple, the mitochondrial membrane potential (ΔΨ), the pH difference across the inner mitochondrial membrane (embedded in the pmf, Δp), and the midpoint potentials of cytochrome c ($E_{mc}$) and cytochrome a3 ($E_{ma}$) in the binuclear center.

$$\begin{array}{cc} v_{c_{4}} & =k_{c_{4}}\cdot a^{2+}\cdot c^{2+}\frac{\left[O_{2}\right]}{\left[O_{2}\right]+K_{m,O_{2}}}\\ V_{T}\cdot \ln\left(\frac{a^{3+}}{a^{2+}}\right) & =\Delta p\left(1+u\right)+E_{mc}+V_{T}\cdot \ln\left(\frac{c^{3+}}{c^{2+}}\right)-E_{ma}\\ u & =\frac{\Delta \Psi }{\Delta p}\\ V_{T} & =\frac{RT}{F}\approx 26\mathrm{mV}\\ E_{mc} & =250mV\\ E_{ma} & =540mV\\ K_{m,O_{2}} & =12\mu M \end{array}$$

Beard’s OXPHOS model [69] used mass action kinetics for cytochrome c and oxygen and included a hyperbolic dependence for the oxygen concentration.

$$\begin{array}{cc} J_{C_{4}} & =X_{C_{4}}\left(\frac{\left[O_{2}\right]}{\left[O_{2}\right]+K_{O_{2}}}\right)\left(\frac{\left[{\mathrm{cytc}}^{2+}\right]}{\left[\sum \mathrm{cytc}\right]}\right)\exp\left(\frac{F\Delta \Psi_{m}}{RT}\right)\left({\left(K_{eq}^{C_{4}}\right)}^{0.5}\left[{\mathrm{cytc}}^{2+}\right]{\left[O_{2}\right]}^{0.25}-\left[{\mathrm{cytc}}^{3+}\right]\right)\\ K_{eq}^{C_{4}} & =\frac{{\left[H^{+}\right]}_{\mathrm{in}}^{4}}{{\left[H^{+}\right]}_{\mathrm{out}}^{2}}\exp\left(-\frac{\Delta_{r}G_{C_{4}}^{0}+4F\Delta \Psi_{m}}{RT}\right) \end{array}$$

The reaction rate of complex IV ($J_{C_{4}}$) depended on the enzyme activity $X_{C_{4}}$, the oxygen concentration ($\left[O_{2}\right]$) and affinity ($K_{O_{2}}$), the cytochrome c pool size ([∑cytc]), the mitochondrial membrane potential ($\Delta \Psi_{m}$), and the apparent equilibrium constant of complex IV ($K_{eq}^{C_{4}}$), which depended on the proton concentration, the $\Delta \Psi_{m}$, and the changes in Gibbs free energy ($\Delta_{r}G_{C_{4}}^{0}$).

Heiske et al. [71] built an OXPHOS model that described the complex IV reaction rate ($v_{c4}$) based on Henry–Michaelis–Menten kinetics.

$$\begin{array}{cc} v_{C_{4}} & =\frac{k_{f}\phi_{c_{2}}\phi_{O_{2}}^{0.25}-k_{b}\phi_{c_{3}}}{\left(1+\phi_{c_{2}}+\phi_{c_{3}}\right){\left(1+\phi_{O_{2}}\right)}^{0.25}}\\ \phi_{c_{3}} & =\frac{\left[c_{3}^{+}\right]}{K_{m}^{c_{3}}},\;\;\phi_{c_{2}}=\frac{\left[c_{2}^{+}\right]}{K_{m}^{c_{2}}}\\ \phi_{O_{2}} & =\frac{\left[O_{2}\right]}{K_{m}^{O_{2}}} \end{array}$$

where $k_{f}$ is the forward reaction constant, $k_{b}$ is the backward reaction constant, and $K_{m}^{x}$ is the apparent Michaelis constant for species *x* (oxygen, reduced and oxidized cytochrome c). The reaction constants and apparent Michaelis constants are thermodynamically constrained by the change in the Gibbs free energy.

**Figure 8:**
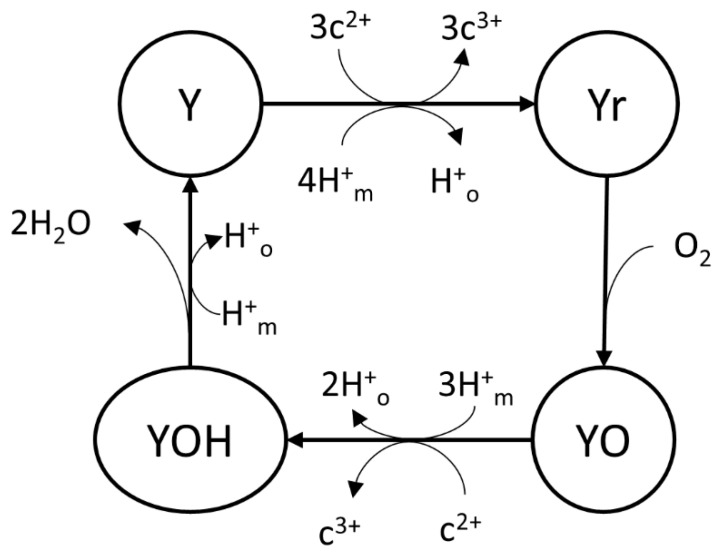
Schematic of the complex IV model by Demin et al. [78] with 4 stages (Y, Yr, YO, and YOH). The figure was reprinted/adapted with permission from Ref. [62].

Demin et al. [78] built a reaction cycle of four states (Y, Yr, YO, and YOH) for complex IV with mass action kinetics as part of their OXPHOS model (Figure 8). The mitochondrial redox balance model by Gauthier et al. [62] used the King–Altman–Hill diagram method on this complex IV model to eliminate intermediate state variables of Y, Yr, YO, and YOH.

The complex IV model by Krab et al. [115] examined changing Michaelis constants for oxygen under different conditions. The 26-step model represented the redox and oxygen binding states of the redox centers. The steady-state oxygen consumption rate was determined by the King–Altman–Hill diagram method. The authors found that the apparent Michaelis constant for oxygen increased under a high pmf or a highly oxidized cytochrome c pool, consistent with experimental results.

Wilson et al. [116] modeled complex IV around the redox states of the binuclear iron-copper center. Between state transitions, the equilibrium constants came from the midpoint potentials of oxygen and binuclear center reduction reactions; the kinetic constants were fitted from experiments on isolated mitochondria. The steady-state oxygen consumption rate came from mass action kinetics. The authors showed that the reaction rates were highly dependent on the energy state Q, acidity, and redox state of the cytochrome c pool. They also revealed that the apparent Michaelis constant for oxygen increases as the energy state Q increases, similar to the results by Krab et al. [115]. 

Pannala et al. [117] improved the complex IV model by Wilson et al. [116], by converting the arbitrary energy state Q to the proton motive force (1 Q = 1.5 pmf), adding competitive and uncompetitive inhibition by nitric oxide (NO), adding a proton pumping mechanism coupled with each cyt c oxidation, assuming all steps are reversible to conform to strict thermodynamic constraints, and deriving steady-state reaction rates from the King–Altman–Hill diagram method. This model reproduced turnover rates under a range of pmf and cyt c redox states, as well as NO inhibition under a range of oxygen levels and cyt c redox states.

## 8. Complex V

OXPHOS complex V, also known as ATP synthase and F1Fo-ATPase, generates magnesium-bound ATP (MgATP) from magnesium-bound ADP (MgADP) and inorganic phosphate (Pi). To make this thermodynamically unfavorable reaction possible, complex V utilizes the proton electrochemical gradient across the inner mitochondrial membrane, the pmf through Peter Mitchell’s chemiosmotic mechanism [3,4,118,119,120].

$${\mathrm{MgADP}}^{-}+H_{2}{\mathrm{PO}}_{4}^{-}+{\mathrm{nH}}_{\mathrm{out}}^{+}\to {\mathrm{MgATP}}^{2-}+H_{2}O+nH_{in}^{+}$$

where n is the number of protons translocated per ATP, also called the H+/ATP ratio, which is approximately three in yeast and mammalian mitochondria [121,122].

### 8.1. Molecular Structure and Reaction Mechanism

Mitochondrial complex V is a membrane-bound molecular motor on the inner mitochondrial membrane (IMM). Complex V can be separated by water solubility into a soluble globular F_1_ catalytic sector in the matrix (subunit α, β, γ, δ, and ɛ) and a membrane-bound F_0_ proton-translocating sector (subunit c-ring, a, b, d, e, f, g, A6L, and oligomycin sensitivity-conferring protein (OSCP)). Mechanically, complex V can be divided into the rotor part (subunit c-ring, γ, δ, and ɛ) and the stator part (the remainder) [120,123,124].

Complex V acts like a turbine generator. The proton gradient across the inner mitochondrial membrane rotates the turbine (oligomeric c ring) and the stalk (gamma subunit) connected to the turbine [124]. In the active site of alpha3/beta3, MgADP and Pi are collected and assembled into MgATP. The mechanical interactions between the rotating stalk and the stator subunits α3/β3 induce a conformational change, altering the binding affinity of MgADP/Pi/MgATP substrates. MgATP is then “pushed” out of complex V, completing the catalytic cycle [120].

Complex V proteins often form a row of V-shaped dimers on the apex of mitochondrial cristae to enhance the efficiency of ATP synthesis. Because proton-pumping ETC complexes concentrate on mitochondrial cristae, the proton gradient is the greatest across the cristae interior and the intermembrane space, allowing efficient ATP synthesis [124,125,126].

Complex V can be inhibited by a range of substances. Oligomycin blocks the proton channels in the c-ring and is commonly used in mitochondrial metabolism studies [123]. 

ATPase inhibitory factor 1 (IF1) is a regulatory protein [123] that “locks” complex V activity during a low mitochondrial membrane potential or acidification of the matrix, preventing cellular ATP consumption in deenergized mitochondria [127,128,129].

Recent studies by Juhaszova et al. [130,131] demonstrated that complex V can also harness the “potassium motive force” to generate ATP in experiments on proteoliposome (PL)-reconstituted purified ATP synthase and isolated mitochondria. The potassium ion influx is driven by the mitochondrial membrane potential and the potassium concentration gradient.

$${\mathrm{MgADP}}^{-}+H_{2}{\mathrm{PO}}_{4}^{-}+{\mathrm{nK}}_{\mathrm{out}}^{+}\to {\mathrm{MgATP}}^{2-}+H_{2}O+nK_{in}^{+}$$

Potassium ions entering the mitochondrial matrix are translocated back out by the mitochondrial K+/H+ exchanger (mKHE), with a 1:1 stoichiometry of protons entering the mitochondrial matrix [132,133].

$$K_{\mathrm{in}}^{+}+H_{\mathrm{out}}^{+}\to H_{\mathrm{in}}^{+}+K_{\mathrm{out}}^{+}$$

Therefore, these study results are fully compatible with Mitchell’s chemiosmotic mechanism.

### 8.2. Kinetic Models of Complex V

In Korzeniewski’s rate skeletal muscle OXPHOS models [53,66,67], the ATP synthesis rate ($J_{C_{5}}$) was nonlinearly dependent on the thermodynamic force (γ) of complex V. The thermodynamic span was the displacement from the thermodynamic equilibrium and was determined by the pmf (Δp), the ratio of ADP and inorganic phosphate to ATP, and the free energy change of ATP synthesis ($\Delta G_{p0}$). The H:ATP ratio ($n_{A}$) was 8/3.

$$\begin{array}{cc} J_{C_{5}} & =k_{C5}\frac{\gamma -1}{\gamma +1}\\ \gamma & =\exp\left(\frac{n_{A}\Delta p\cdot F-\Delta G_{p0}}{RT}\right)\cdot \frac{{\left[\mathrm{ADP}\right]}_{m}\cdot {\left[\mathrm{Pi}\right]}_{m}}{1M\cdot {\left[\mathrm{ATP}\right]}_{m}} \end{array}$$

Beard’s OXPHOS model [69] assumed that complex V operated close to equilibrium and utilized mass action kinetics for its substrates: ATP, ADP, and inorganic phosphate.

$$\begin{array}{cc} J_{C5} & =X_{F_{1}}\left(K_{eq}^{F_{1}}{\left[\mathrm{ADP}\right]}_{m}{\left[\mathrm{Pi}\right]}_{m}-{\left[\mathrm{ATP}\right]}_{m}\right)\\ K_{eq}^{F_{1}} & =\frac{{\left[H^{+}\right]}_{\mathrm{out}}^{3}}{{\left[H^{+}\right]}_{\mathrm{in}}^{2}}\cdot \frac{P_{ATP}}{P_{ADP}P_{PI}}\exp\left(-\frac{\Delta_{r}G_{F_{1}}^{0}-3F\Delta \Psi_{m}}{RT}\right) \end{array}$$

where $X_{F_{1}}$ is the activity of complex V, and $K_{eq}^{F_{1}}$ is the apparent equilibrium constant depending on the proton gradient, binding polynomials of substrates ($P_{ATP}$, $P_{ADP}$, $P_{PI}$), change in Gibb’s free energy ($\Delta_{r}G_{F_{1}}^{0}$), and the mitochondrial membrane potential ($\Delta \Psi_{m}$).

Heiske et al. [71] built an OXPHOS model that described the complex V reaction rate ($J_{c5}$) based on Henry–Michaelis–Menten kinetics.

$$\begin{array}{cc} J_{C_{5}} & =\frac{k_{f}\phi_{D}\phi_{P}-k_{b}\phi_{T}}{\left(1+\phi_{D}+\phi_{T}\right)\left(1+\phi_{P}\right)}\\ \phi_{D} & ={\left[\mathrm{ADP}\right]}_{m}/K_{ADP}\\ \phi_{T} & ={\left[\mathrm{ATP}\right]}_{m}/K_{ATP}\\ \phi_{P} & ={\left[\mathrm{Pi}\right]}_{m}/K_{Pi} \end{array}$$

where $k_{f}$ is the forward reaction constant, $k_{b}$ is the backwards reaction constant, and $K_{m}^{x}$ is the apparent Michaelis constant for species *x* (ATP, ADP, inorganic phosphate). The reaction constants and apparent Michaelis constants are thermodynamically constrained by the change in the Gibbs free energy.

The beta cell mitochondria model by Magnus and Keizer [54] assumed that complex V was a six-state proton pump operating in reverse, using the same framework as the ETC in the same model. The ATP synthesis rate and the proton consumption rate strongly depended on both the mitochondrial membrane potential and the phosphorylation potential. The H^+^/ATP stoichiometry was higher than 3:1 due to the presence of slip reactions.

$$\begin{array}{cc} V_{\mathrm{ATPase}\;} & =-\rho^{F1}\frac{\left(10^{3\Delta pH}p_{a}+p_{c1}f_{B}+p_{c2}f_{m}\right)f_{A}-p_{a}f_{m}}{\left(1+p_{1}f_{A}\right)f_{B}+\left(p_{2}+p_{3}f_{A}\right)f_{m}}\\ V_{Hu} & =-3\rho^{F1}\frac{10^{3\Delta pH}p_{a}f_{A}-\left(p_{a}+p_{b}\right)f_{m}}{\left(1+p_{1}f_{A}\right)f_{B}+\left(p_{2}+p_{3}f_{A}\right)f_{m}}\\ f_{A} & =\frac{K_{eq}^{F1}{\left[ATP\right]}_{m}}{{\left[Pi\right]}_{m}{\left[ADP\right]}_{m}}\\ f_{B} & =\exp\left(3\Delta \Psi_{B}/V_{T}\right)\\ f_{m} & =\exp\left(3\Delta \Psi_{m}/V_{T}\right)\\ \Delta pH & =pH_{i}-pH_{m}\\ V_{T} & =\frac{RT}{F} \end{array}$$

where $V_{ATPase}$ is the ATP generation rate, $V_{Hu}$ is the proton flux through complex V, $V_{T}$ is the thermal voltage, $\rho^{F1}$ is the concentration of complex V, $K_{eq}^{F1}$ is the apparent equilibrium constant, $\Delta \Psi_{B}$ is the phase boundary potential, and $p_{a}$, $p_{b}$, $p_{c1}$, $p_{c2}$, $p_{1}$, $p_{2}$, and $p_{3}$ are the parameters of complex V.

**Figure 9:**
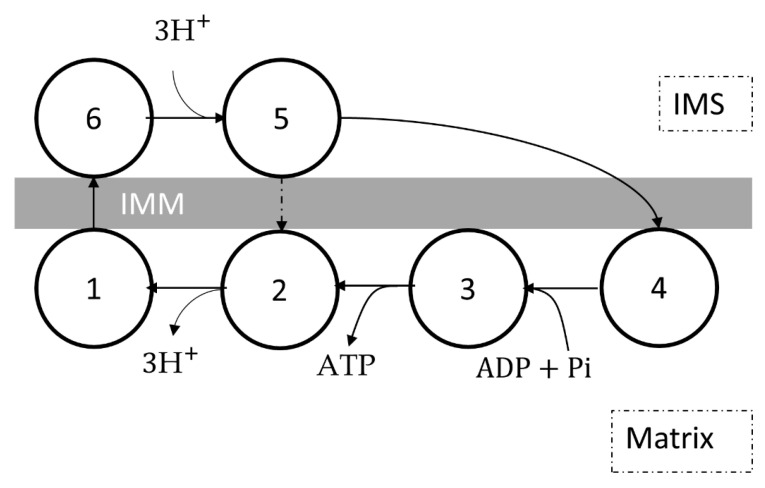
Schematic of the complex V model by Magnus and Keizer [54], assuming complex V is a 6-state proton pump operating in reverse. Not accounting for slip reactions (5 → 2), one ATP molecule is generated for every 3 protons translocated from the intermembrane space (IMS) to the matrix. The figure was reprinted/adapted from Magnus and Keizer [54].

The mitochondrial energetics model for guinea pig ventricular cardiomyocytes by Cortassa et al. [56] replaced the dependence on the mitochondrial membrane potential with the dependence on the pmf in Magnus and Keizer’s model (Figure 9). A factor of 100 was also added to address reversibility under low pmf. Later models [134,135,136,137,138,139] also utilized this schematic for mitochondrial energetics in guinea pig ventricular cardiomyocytes.

$$\begin{array}{cc} V_{\mathrm{ATPase}\;} & =-\rho^{F1}\frac{\left(100p_{a}+p_{c1}v_{B}+p_{c2}v_{H}\right)v_{A}-p_{a}v_{H}}{\left(1+p_{1}v_{A}\right)v_{B}+\left(p_{2}+p_{3}v_{A}\right)v_{H}}\\ V_{Hu} & =-3\rho^{F1}\frac{100p_{a}\left(1+v_{A}\right)-\left(p_{a}+p_{b}\right)v_{H}}{\left(1+p_{1}v_{A}\right)v_{B}+\left(p_{2}+p_{3}v_{A}\right)v_{H}}\\ v_{A} & =\frac{K_{eq}^{F1}{\left[ATP\right]}_{m}}{{\left[Pi\right]}_{m}{\left[ADP\right]}_{m}}\\ v_{B} & =\exp\left(3\Delta \Psi_{B}/V_{T}\right)\\ v_{H} & =\exp\left(3\Delta p/V_{T}\right)\\ \Delta p & =\Delta \Psi_{m}-2.303V_{T}\cdot \Delta pH\\ \Delta pH & =pH_{i}-pH_{m}\\ V_{T} & =RT/F \end{array}$$

The complex V model by Cortassa et al. in 2022 [140] incorporated ATP generation from the potassium gradient, based on the studies of Juhaszova et al. [130,131]. The mathematical descriptions were based on the model of Magnus and Keizer [54] and Wei et al. [77]. In addition to the proton flux ($J_{C_{5}}^{H}$), the model also considered the potassium ($J_{C_{5}}^{K}$) and the sodium fluxes ($J_{C_{5}}^{Na}$) through complex V. All three fluxes contributed to ATP synthesis ($J_{C_{5}}^{ATP}$).

$$\begin{array}{cc} J_{C_{5}}^{ATP} & =-\rho^{F1}\left(\left(p_{a}f_{MCa}+p_{c1}v_{B}\right)v_{A}-\left(p_{a}+p_{c2}v_{A}\right)v_{I}\right)/\Delta \\ J_{C_{5}}^{H} & =-3\rho^{F1}\left(10^{-3\Delta \mathrm{pH}}p_{a}v_{A}-\left(p_{a}+p_{b}\right)v_{H}\right)/\Delta \\ J_{C_{5}}^{K} & =-3\rho^{F1}f_{K}\left(10^{-3\Delta \mathrm{pK}}p_{a}v_{A}-\left(p_{a}+p_{b}\right)v_{K}\right)/\Delta \\ J_{C_{5}}^{Na} & =-3\rho^{F1}f_{Na}\left(10^{-3\Delta \mathrm{pNa}}p_{a}v_{A}-\left(p_{a}+p_{b}\right)v_{Na}\right)/\Delta \\ \Delta & =\left(1+p_{1}v_{A}\right)v_{B}+\left(p_{2}+p_{3}v_{A}\right)v_{I}\\ v_{B} & =\exp\left(3\Delta \Psi_{B}/V_{T}\right)\\ v_{H} & =\exp\left(3\Delta \mu_{H}/V_{T}\right)\\ v_{K} & =\exp\left(3\Delta \mu_{K}/V_{T}\right)\\ v_{Na} & =\exp\left(3\Delta \mu_{Na}/V_{T}\right)\\ v_{I} & =\exp\left(3\left(\Delta \mu_{H}+\Delta \mu_{K}+\Delta \mu_{Na}\right)/V_{T}\right)=v_{H}v_{K}v_{Na}\\ v_{A} & =\frac{K_{\ell q}^{F_{1}}{\left[{\mathrm{MgATP}}^{2-}\right]}_{m}}{\left[{\mathrm{Pi}}_{m}{\left[\mathrm{ADP}\right]}_{m}^{\mathrm{free}\;}\right.}\\ f_{MCa} & =10^{3\Delta \mathrm{pH}}+f_{K}10^{3\Delta \mathrm{pK}}+f_{Na}10^{3\Delta \mathrm{pNa}}\\ \Delta \mu_{H} & =\Delta \Psi_{m}+Z\Delta \mathrm{pH}\\ \Delta \mu_{K} & =\Delta \Psi_{m}+Z\Delta \mathrm{pK}\\ \Delta \mu_{Na} & =\Delta \Psi_{m}+Z\Delta \mathrm{pNa}\\ Z & =2.303V_{T}\\ V_{T} & =RT/F\\ \Delta \mathrm{pH} & ={\mathrm{pH}}_{i}-{\mathrm{pH}}_{m}\\ \Delta \mathrm{pK} & ={\mathrm{pK}}_{i}-{\mathrm{pK}}_{m}\\ \Delta \mathrm{pNa} & ={\mathrm{pNa}}_{i}-{\mathrm{pNa}}_{m} \end{array}$$

The complex V descriptors in Nguyen et al.’s cardiomyocyte OXPHOS model [141] used Hill kinetics for the mitochondrial membrane potential and random-order Bi-Uni kinetics for ADP, ATP, and inorganic phosphate (Pi). The calcium activation of ATP synthesis was also considered. The complex V reaction was assumed to be fully coupled; thus, the H:ATP ratio was exactly 3:1.

$$\begin{array}{cc} V_{C_{5}} & =\frac{V_{max}\left(f_{D}f_{P}-f_{T}\right)f_{v}f_{c}}{1+f_{D}+f_{P}+f_{T}+f_{D}f_{P}}\\ f_{D} & =\left[ADP\right]/K_{ADP}\\ f_{T} & =\left[ATP\right]/K_{ATP}\\ f_{P} & =\left[Pi\right]/K_{Pi}\\ f_{v} & =\frac{\Delta \Psi_{m}^{8}}{K_{v}^{8}+\Delta \Psi_{m}^{8}}\\ f_{c} & =1-\exp\left(-{\left[{\mathrm{Ca}}^{2+}\right]}_{m}/K_{Ca}\right) \end{array}$$

where $V_{C_{5}}$ is the ATP synthesis rate, $V_{max}$ is the activity of complex V, $K_{ATP}$, $K_{ADP}$, and $K_{Pi}$ are the dissociation constants for ATP, ADP, and inorganic phosphate, respectively; $K_{v}$ is the voltage activation factor, and $K_{Ca}$ is the calcium activation factor.

The complex V model by Metelkin et al. [79,142] to study the interactions between complex V and the adenine nucleotide translocator (ANT) used a similar scheme and included the concentrations of protons on both sides of the IMM.

$$\begin{array}{cc} V_{C_{5}} & =V_{\max}^{C5}{\left(\frac{H_{o}}{K_{H_{o}}}\exp\left(\chi \phi \right)\right)}^{n}\frac{N}{Den}\\ N & =f_{D}f_{P}-f_{T}K_{eq}^{\prime }{\left(\frac{H_{o}}{H_{i}}\exp\left(\phi \right)\right)}^{-n}\\ \;\mathrm{Den}\; & =1+f_{D}f_{P}f_{H_{o}}+f_{T}f_{H_{i}}\exp\left(-n\left(1-\chi \right)\phi \right)\\ K_{eq}^{\prime } & =K_{eq}^{C5}\frac{K_{MgT}^{C5}K_{Mg}^{ATP}}{K_{MgD}^{C5}K_{Pi}^{C5}K_{Mg}^{ADP}}\cdot \frac{10^{-7}M}{10^{-7}M+K_{P,H}}\\ f_{D} & =\left[MgADP\right]/K_{MgD}^{C5}\\ f_{T} & =\left[MgATP\right]/K_{MgT}^{C5}\\ f_{P} & =\left[Pi\right]/K_{Pi}^{C5}\\ f_{H_{o}} & ={\left(\frac{H_{o}}{K_{H_{o}}^{C5}}\right)}^{n}\\ f_{H_{o}} & ={\left(\frac{H_{i}}{K_{H_{i}}^{C5}}\right)}^{n}\\ \phi & =\frac{F\Delta \Psi_{m}}{RT} \end{array}$$

## 9. Summaries of the OXPHOS Models 

In 1967 Chance proposed the first OXPHOS model using operational flux expressions [143], Bohnensack [144] and Holzhutter [145] later constructed the first kinetic OXPHOS model and used thermodynamic force to drive the OXPHOS reactions [146]. These studies have established the foundation of kinetic modeling of OXPHOS, and more detailed and sophisticated models have been proposed since then [146]. In this section, we summarize the mitochondrial OXPHOS computational modeling mentioned in the previous sections in Table 1. Because many of the studies included two or more respiratory complexes, we provide overall remarks in this section rather than in the respective OXPHOS complex sections above.

The rat hepatocyte [52,114] and skeletal muscle cell OXPHOS models [53,67] by Korzeniewski et al. included OXPHOS complexes I, III, IV, and V, along with proton leak, substrate dehydrogenation, adenine nucleotide translocator (ANT), phosphate carrier (PiC), and adenylate kinase (AdK). Cytochrome c reduction (by complex I and complex III) and ATP synthesis (by complex V) rates were linked to their thermodynamic spans, which is the displacement from thermodynamic equilibria. Complex I, III, and V reactions were assumed to operate near equilibrium [147]. Cytochrome c oxidation (by complex IV) used mass action kinetics. The oxygen-binding reduced heme a3 was assumed to be in rapid equilibrium and derived by oxidized to reduced cytochrome c ratio and the pmf. Korzeniewski et al. used these models to study the metabolic flux control of ATP generation and consumption in different metabolic modes (glycolysis, fatty acid oxidation, and gluconeogenesis) in hepatocytes [114,148], metabolic flux control and the impact of respiratory complex deficiencies in skeletal muscle cells [53], comparing the negative feedback and parallel activation mechanisms of ATP synthesis and consumption in skeletal muscle cells [66,67,149], and the regulation of OXPHOS reactions in cardiomyocytes [68]. They demonstrated that parallel activation to NADH generation, proton pumping by respiratory complexes, ATP synthesis, and ATP consumption could explain why the ATP to ADP ratio barely changes when the work intensity increases. The OXPHOS models by Korzeniewski et al. are simple, thermodynamically consistent (for complexes I, III, V, and ANT), and in good agreement with experimental data of ATP synthesis, proton leak, and oxygen consumption. However, they did not include detailed descriptions of the citric acid cycle, calcium dynamics, and ROS generation/scavenging mechanisms.

Saito et al. [150] built a cardiomyocyte mitochondrial model, which integrated complex I, III, IV, and V models by Korzeniewski et al. [52,53,67,114], citric acid cycle reactions, ion (proton, sodium, calcium, potassium, and phosphate) and respiring substrate (malate and glutamate) dynamics, and high energy phosphate buffering by adenylate kinase and creatine kinase. The authors used this model to study why the metabolite levels of NADH and ATP remain relatively stable under various workloads. They found that inorganic phosphate (Pi) stimulated ATP synthesis more effectively than calcium.

Beard’s OXPHOS model [69] included complexes I, III, IV, and V, as well as ANT, PiC, AdK, potassium leakage, and potassium efflux through mKHE. The reaction rates of the OXPHOS complexes were described by mass action kinetics, assuming they operated near equilibrium. The reaction rate constants were also constrained by the change of Gibbs free energy in each complex and fitted by the Bose et al. dataset [70] of porcine heart and skeletal muscle mitochondria. However, this mitochondrial model did not include explicit citric acid cycle processes, ROS generation/scavenging, or mitochondrial calcium dynamics. This model required complex III to be explicitly activated by inorganic phosphate to fit the experimental data. The simple mass action kinetics descriptors did not follow saturation kinetics in enzyme-catalyzed reactions.

Wu et al. [82] extended Beard’s OXPHOS model [69] and integrated citric acid cycle reactions (including complex II, succinate dehydrogenase) and substrate exchange across the IMM. The parameters were estimated based on the Bose et al. dataset [70] of porcine heart and skeletal muscle mitochondria and the LaNoue et al. dataset [151] of rat heart mitochondria. The authors concluded that ADP and NAD regulated the citric acid cycle fluxes. In contrast, ATP synthesis was regulated by inorganic phosphate, which activated complexes III and V. However, ROS generation/scavenging and calcium dynamics were not included in this model. Bazil et al. [83] extended the mitochondrial model by Wu et al. [82] and integrated sodium, calcium, and potassium influx/efflux reactions as well as mitochondrial matrix volume dynamics due to osmolarity changes caused by the matrix potassium concentration.

Heiske et al. [71] built an OXPHOS model based on the OXPHOS models of Beard [69] and Korzeniewski and Zoladz [67]. The authors used Henry–Michaelis–Menten kinetics instead of mass action kinetics for the rate equations of the OXPHOS complexes (I, III, IV, V) to address saturation in enzyme kinetics. The parameters were constrained by the changes in the Gibbs free energy and fitted by their experimental data [71,152] and the Bose et al. dataset [70]. However, this model did not include citric acid cycle reactions, ROS generation and scavenging, and calcium dynamics.

The complex III monomer [98] and dimer [99] model by Bazil et al., the complex I model [61] by Bazil et al., and the complex II model by Manhas et al. [84] have used quasi-steady state assumptions to describe the overall reaction rates of substrate consumption and ROS formation. Each model had several redox states representing the number of electrons in the redox centers. Redox reactions between the complex and substrates were state transitions. The kinetic parameters were constrained by Gibbs free energy changes of the reactions and fitted by various datasets, primarily from bovine cardiomyocyte mitochondria experiments. By applying the quasi-steady state assumption and setting the rate of change of each state to zero, the overall reaction rates could be solved from a system of linear equations of states and their transition rates. These respiratory complex models have reproduced redox reaction rates under various conditions. They have been used to study the source of ROS generation in ischemia/reperfusion (I/R) injury [81,153] and to test the hypothesis of calcium phosphate inhibiting complex I during calcium overload in a computational-experimental approach [154]. 

Markevich et al. built complex I [63], III [63], and II [85] models using mass action kinetics. These models have been used to study ROS generation mechanisms and steady-state ROS production rates in various substrate inputs and a range of mitochondrial membrane potentials. Because their mass action kinetics models included detailed reaction steps of substrate binding, proton pumping, enzyme state transitions, and ROS generation, the authors can test different ROS generation mechanisms and simulate complex inhibition by changing the kinetic constants.

The minimal mitochondrial model by Magnus and Keizer [54] included a lumped respiratory chain (complex I-III-IV) and ATP synthase (complex V) for the OXPHOS system as well as adenine ANT, calcium influx via the mitochondrial calcium uniporter (MCU), and calcium efflux via the mitochondrial sodium-calcium exchanger (mNCX). Matrix proton, inorganic phosphate, and sodium concentrations were assumed to be constant, so their dynamics were not included. ROS production and scavenging systems were also absent. The OXPHOS system used the six-state proton pump scheme from Pietrobon and Caplan [55]. The (quasi-)steady-state rates of NADH oxidation, proton translocation, and ATP synthesis were derived by the King–Altman–Hill diagram method [50]. Magnus and Keizer later incorporated this mitochondrial model with cellular glycolysis and ion channels to simulate the mouse beta-cell membrane potential, ATP, and calcium oscillations in the presence of glucose [155,156].

The mitochondrial energetics model by Cortassa et al. [56] integrated the calcium dynamics and OXPHOS from the Magnus–Keizer model [54] and the citric acid cycle (CAC) reactions from the Jafri–Dudycha model [157]. The CAC provided NADH for the ETC and was stimulated by mitochondrial calcium. The model was used to simulate the generation and consumption of NADH and ATP in cardiomyocyte mitochondria under various workloads, demonstrating that mitochondrial calcium can enhance energy supply. However, the succinate oxidation in the respiratory chain did not couple with the succinate dehydrogenase reaction in the CAC. Later the same group integrated the mitochondrial model with electrophysiology and muscle contraction systems and constructed the excitation-contraction coupling/mitochondrial energetics (ECME) model of guinea pig ventricular cardiomyocytes [136]. The simulations were in good agreement with the experimental results of force-frequency relationships, mitochondrial NADH, and mitochondrial calcium changes in response to varying workloads.

Bertram et al. [158] simplified Cortassa et al.’s model [56], known as the BLPS model, to simulate mitochondrial ATP synthesis. The electron transport chain and ATP synthase were described by Michaelis–Menten kinetics for the substrates (NADH, ADP) and sigmoid dependence on the mitochondrial membrane potential. Saa and Siqueira [159] improved and corrected the BLPS model to study the metabolic response under a range of glycolysis fluxes and oscillatory calcium levels.

Nguyen et al. [141] built a cardiomyocyte mitochondrial model that integrated the ETC model from Magnus and Keizer [54], the CAC reactions from the Jafri–Dudycha model [157], and the calcium uniporter and ATP synthase model from the authors’ previous model [160]. The authors added NCX, NHX, and PiC to simulate inorganic phosphate, sodium, and proton dynamics. The models can reproduce mitochondrial calcium concentrations and influx under periodic stimulation, consistent with cardiomyocyte experiments. They also showed that increasing cytosolic sodium levels enhanced calcium extrusion from the mitochondria via the NCX, leading to decreased mitochondrial calcium concentrations and ATP synthesis.

Cortassa et al. [135] built a ROS-induced ROS release (RIRR) model based on their previous model [56]. The authors integrated CAC, OXPHOS, ROS production, ROS-inducible anion channels for ROS efflux, and ROS scavenging by catalase, SOD, and the glutathione (GSH) system. Since there were no mechanistic descriptions for ROS production, it was modeled by taking an adjustable fraction of oxygen consumption to produce superoxide. They simulated the ROS-induced ROS release (RIRR) phenomenon and reproduced mitochondrial membrane potential depolarization and recovery events observed in guinea pig ventricular cardiomyocytes under oxidative stress [161].

Zhou et al. [138] incorporated the ECME model [136] and the RIRR model [135] to study action potential shortening and insensitivity to electrical stimuli due to oxidative stress-induced mitochondrial depolarization, ATP synthase reversal, decreased ATP to ADP ratio, and the opening of ATP-sensitive potassium channels. The ECME-RIRR model has been used to study potential oscillations in a mitochondrial network [137,162], arrhythmogenesis from blocked action potential propagation [139], and abnormal action potential patterns due to ROS disrupting calcium dynamics in the cytosol [163,164].

Wei et al. [77] extended the mitochondrial energetics model by Cortassa et al. [56] by integrating proton, phosphate, sodium, and calcium dynamics. The authors included mathematical descriptors for PiC, MCU, and mNCX. The authors also improved the complex II model by coupling succinate oxidation in the ETC and the CAC. The model results were in good agreement with mitochondrial membrane potential, mitochondrial NADH, and pH changes in guinea pig cardiomyocyte experiments. However, ROS production and scavenging mechanisms were not included in this model. 

Kembro et al. [14] extended the mitochondrial model of Wei et al. [77] and the RIRR model of Cortassa et al. [135] to build a two-compartment (cytosol and mitochondria) ROS production, transport, and scavenging model. The mitochondrial energetic-redox (ME-R) model included mitochondrial NADPH generation from transhydrogenase (THD) and NADPH-producing isocitrate dehydrogenase (IDH2), ROS efflux via anion channels (for superoxide) and simple diffusion (for hydrogen peroxide), and ROS scavenging by catalase, SOD, and the GSH and Trx systems [15]. The fatty acid oxidation model by Cortassa et al. [165] added beta oxidation steps for palmitate to the two-compartment ROS generation, transfer, and scavenging model by Kembro et al. [14] The experimental-computational approach showed that ROS excess upon palmitate addition resulted from impaired GSH and Trx ROS scavenging systems and palmitate-induced uncoupling. 

Cortassa et al. [140] extended the model of Kembro et al. [14] by including a complex V model utilizing the potassium gradient to generate ATP and an ETC model coupled to mitochondrial matrix volume dynamics, which was controlled by potassium content. The authors reproduced experimental data of changes in matrix volume, potassium uptake, and mitochondrial membrane potential from low to high energy demand.

Gauthier et al. [62] built a mitochondrial model, called the ETC-ROS model, to study the redox balance hypothesis [166], which stated that ROS production is minimal and energy generation is maximal when the cellular environment is moderately reduced. Thus, a mechanism of ROS generation from the respiratory complexes was needed. The complex I model was derived from the six-state proton pump scheme of Pietrobon and Caplan [55]. The complex I model included an oxygen-reducing pathway for superoxide production and an additional state for ubiquinone reduction. It reproduced the guinea pig cardiomyocyte mitochondrial data of Aon et al. [166]. The complex II, III, and IV models were modified from the hepatocyte mitochondrial model by Demin et al. [78,79] to reproduce the macrophage OXPHOS data from Kim et al. [167]. The complex V model was based on the mitochondrial model by Wei et al. [77]. The ROS scavenging systems included the SOD, GSH, and Trx systems. The authors showed that mitochondrial ROS production was minimized when the redox environment (NAD/NADH and NADP/NADPH couples) was moderately reduced, supporting the redox-optimized ROS balance (R-ORB) hypothesis [166].

Later Gauthier et al. reported a new mitochondria model [72] that incorporated the OXPHOS and ROS generation parts from the ETC-ROS model [62] and the ROS scavenging parts from the ME-R model [14] to study ROS production in heart failure. Their model can reproduce respiration rates, NADH to NAD ratios, and ROS production rates at state three (ADP present) and state four (ADP absent). The model also demonstrated that either blocking mitochondrial calcium uniporter or increasing cytosolic sodium concentrations (as in heart failure) suppressed mitochondrial calcium, NADH, NADPH, and GSH levels while ROS production increased. De Oliveira et al. [168] extended this model [72] and included doxorubicin-induced acute and chronic cardiac toxicity. Their model can simulate acute toxicity by inactivating OXPHOS complexes and enhancing ROS production. In contrast, chronic toxicity was simulated by ROS damaging mitochondrial DNA (mtDNA), inhibiting the long-term activity of OXPHOS complexes and leading to a vicious cycle as faulty OXPHOS complexes tend to produce more ROS and damage mtDNA even further. The authors predicted the dose response of both acute and chronic doxorubicin toxicity. They also showed that extending the duration of the iron chelator therapy partially mitigated chronic doxorubicin toxicity and protected cardiomyocytes against mitochondrial dysfunction.

**Table 1:** List of published OXPHOS kinetic models reviewed in this paper.

Model	Respiratory Complex	Cell Type	Method	Internal State Variables
Korzeniewski, 1991 [52]	I-III IV V	Rat hepatocytes	TS MAK TS	0 2 0
Korzeniewski, 1996 [114]	IV	Rat hepatocytes	MAK	0
Korzeniewski, 1996 [53]	I, III	Rat skeletal muscle cells	TS	0
Magnus and Keizer, 1997 [54]	I-III-IV V	Mouse pancreatic beta-cells	KAH KAH	0 0
Cortassa, 2003 [56]	I-III-IV II-III-IV V	Guinea pig ventricular cardiomyocytes	KAH KAH KAH	0 0 0
Saa, 2013 [159]	I-III-IV V	Not specified	Phenomenal Phenomenal	0 0
Beard, 2005 [69]	I, III, IV, V	Porcine heart and skeletal muscle cells	MAK	0
Wu, 2007 [82]	II	Porcine heart and skeletal muscle cells and rat cardiomyocytes	Theorell–Chance Bi-Bi mechanism	0
Heiske, 2017 [71]	I, III, IV, V	Bovine cardiomyocytes	MM	0
Markevich and Hoek, 2015 [63]	I III	Bovine cardiomyocytes	MAK	12 14
Gauthier, 2013 [62]	I III	Guinea pig ventricular cardiomyocytes	KAH MAK	0 10
Bazil et al., 2014 [61]	I	Bovine cardiomyocytes and rat cardiomyocytes	HKA	0
Manhas et al., 2020 [84]	II	Bovine/Pig/Guinea pig cardiomyocytes	KAH	0
Markevich et al., 2020 [85]	II	Bovine and rat cardiomyocytes	MAK	35
Demin et al., 1998 [79]	III	Not specified	MAK	9 (minimal)/12 (channeled)
Demin et al., 2001 [78]	III IV	Hepatocyte	MAK MAK	12 4
Selivanov et al., 2009 [96]	III	Rat brain mitochondria	MAK	400
Guillaud et al., 2014 [97]	III	Rat skeletal muscle, heart, liver, kidney, and brain cells	MAK	16
Bazil et al., 2013 [98]	III	Bovine cardiomyocytes	KAH	0
Krab et al., 2011 [115]	IV	Bovine cardiomyocytes	KAH	0
Wilson et al., 2014 [169]	IV	Rat hepatocytes	Quasi-steady state from MAK	0
Pannala et al., 2016 [117]	IV	Rat hepatocytes and bovine cardiomyocytes	KAH	0
Nguyen et al., 2007 [141]	V	Guinea pig ventricular cardiomyocytes	Hill kinetics for MMP, Random Bi-Uni kinetics for substrate	0
Cortassa et al., 2022 [140]	V	Guinea pig ventricular cardiomyocytes	KAH	0

MAK: mass action kinetics. KAH: King–Altman–Hill diagram method. MM: Michaelis–Menten kinetics. TS: thermodynamic span.

## 10. Other OXPHOS Aspects and Concluding Remarks

In this review, we focus on the mathematical modeling of the OXPHOS complexes, how their reaction mechanisms have been described, and where and how ROS come from. Although we cover the descriptors from complex I to complex V (Table 1), to build an integrated mitochondrial bioenergetics model, additional aspects related to OXPHOS must be addressed.

For instance, ATP generation can be stimulated by mitochondrial calcium, ADP, and inorganic phosphate to meet varying workload demands. Mitochondrial calcium levels increase with cytosolic calcium levels upon excitation-contraction coupling and stimulate dehydrogenases in the CAC. ADP and inorganic phosphate are substrates of complex V to synthesize ATP. Takeuchi et al. [40] conducted a comprehensive review of modeling the tight regulation of ATP levels via calcium, ADP, and inorganic phosphate dynamics. 

ROS generation and scavenging regulation is another important topic in mitochondrial OXPHOS modeling. ROS play a crucial role in physiological metabolic signaling and pathological oxidative stress. Pereira et al. [170] and Mazat et al. [171] discussed the mathematical ETC models of ROS production that could be further extended in studies of oxidative stress and signaling. For instance, Kembro et al. [14] built a two-compartment (cytosolic and mitochondrial) model integrating ROS generation from the ETC, ROS scavenging by SOD, catalase, the GSH and the Trx systems, NADPH production, and superoxide-induced mitochondrial depolarization. The authors found that mitochondrial membrane potential oscillation frequencies and amplitudes were dependent on the counteracting forces of ROS production and the antioxidant capacity [172].

Mitochondrial OXPHOS coordinates with other mitochondrial components to regulate ATP production, transportation, and utilization. ATP generated from complex V must be exported from the mitochondrial matrix to the cytosol via ANT, which catalyzes the 1:1 exchange of free ATP to free ADP across the IMM [142,173].

The creatine kinase (CK) system shuttles and buffers ATP synthesis from the OXPHOS and consumption by biological processes, exchanging high energy phosphates between ATP/ADP and creatine/creatine phosphate couples [174]. Mitochondrial CK (mtCK) couples functionally and structurally with ANT to channel ADP recycling, enhance ATP synthesis, and reduce ROS generation [175,176,177]. Mathematical modeling has shown that mtCK can maintain cellular homeostasis and dampen mitochondrial metabolic oscillation [178].

The ETC is fueled by the CAC. CAC-generated NADH is oxidized in complex I and the CAC intermediate succinate is oxidized in complex II. CAC is coupled with carbohydrate (glycolysis), lipid (beta-oxidation) and amino acid metabolism. Some models keep these inputs as constant parameters [56] for simplicity, while others study the impact of varying fuel inputs. For instance, in a glucose-sensing beta cell model by Fridlyand et al. [179], glycolysis flux can enhance CAC, ETC, and mitochondrial ATP synthesis fluxes and induce insulin secretion. A cardiomyocyte mitochondrial model by Cortassa et al. [165] showed that excessive fatty acids can lead to increased ROS production and mitochondrial uncoupling, both computationally and experimentally.

The ETC architecture in vivo could be more complex than the homogeneous assumption in the ODE-based mathematical models. Respiratory complexes I, III dimer, and IV could assemble into a supercomplex, channeling the electron carriers of ubiquinone and cytochrome c between the complexes to increase efficiency, which may deviate from the pool behavior assumed in ODE models [1,180,181,182]. The ETC complexes have also been shown to concentrate on the cristae membrane, the infoldings of the inner mitochondrial membrane, with complex V (ATP synthase) dimers sitting on the apex of the cristae. The membrane potential was shown to be higher across the crista IMM than across the non-crista IMM. Each crista can hold different membrane potentials, acting as independent ATP-generating units [125].

To date, several integrative cellular models have been developed to study the interactions of mitochondrial energetics and the cellular environment. Computational models have been used to study the energy supply and demand in cardiomyocytes [37,136,177,183], glucose sensing and insulin secretion in pancreatic beta cells [156,179], metabolic interactions between neurons and astrocytes in the brain [184,185,186], hepatocellular respiration in the liver [187], and metabolic adaptation during exercise in the skeletal muscle [188,189]. Due to tissue differences in energetics demands, mitochondrial distributions and functions are very different in each tissue [190]. To consider mitochondrial energetics, the model parameters must be adjusted and validated by tissue-specific data. The mitochondria and OXPHOS model could also apply to heart failure studies or iPSC studies in patients. For example, induced pluripotent stem cell-derived cardiomyocytes (iPSC-CMs) provide a tool for disease modeling, including mitochondrial cardiomyopathy and metabolic disorders with cardiac phenotypes [191]. Cardiac computational modeling [192,193] with bioenergetics details can provide insight into the mechanisms in disease development. 

In this review, we focus on deterministic kinetic models of OXPHOS, which emphasize the dynamic, continuous, and time-dependent interactions of each complex and substrate. Stochastic models of the ETC complex that focus on discrete events of electron transfer within a complex are also available [65,90,194,195]. While these models provide more details for the underlying molecular mechanism than deterministic kinetic models, they require more simulation time than deterministic models. They are more challenging to integrate with other modeling components. Another way to build OXPHOS ATP generation models is constraint-based modeling (CBM), which considers biochemical reactions as a whole and uses a stoichiometric matrix of metabolites and reactions to deduce the best plausible steady-state target flux (e.g., ATP synthesis rate) under some constraints in the reaction fluxes [196]. Although CBM requires far less parameters to fit and could handle genome-scale metabolic model (GEMMs) containing thousands of reactions and hundreds of metabolites, CBM cannot capture transient dynamic responses and complex interactions between components in the same way as kinetic models [197].

In conclusion, we review various mathematical kinetic modeling strategies for OXPHOS complexes. The OXPHOS is a highly nonlinear system with complicated time-variant responses on a range of inputs, such as respiring substrates, ions (including protons, phosphate, magnesium, calcium, sodium, and potassium), and the mitochondrial membrane potential. Lumped models require the least number of parameters and intermediate state variables but omit some mechanistic details. Mass action kinetic models give the most detailed inner workings but include many parameters that must be fitted and intermediate state variables that must be computed. Quasi-steady state kinetics models for individual complexes sit in between the two. The OXPHOS system is also interconnected to other mitochondrial bioenergetics systems, as represented mathematically by their inputs: dependence on respiring substrate concentration and the pmf, and by their outputs: generating ATP, pmf, and ROS. We also briefly discuss other bioenergetics topics not covered in detail, such as the CAC, ATP export, the creatine shuttle, ROS production/scavenging, and tissue/species differences. These OXPHOS modeling strategies and other interconnected systems can help us to build an integrative bioenergetics model for cellular metabolism.

## Data Availability

Not applicable.
